# Supplementary material for: Phytochemical Screening and Characterization of Volatile Compounds from Three Medicinal Plants with Reported Anticancer Properties Using GC-MS
Source: Life (Basel). 2024 Oct 25;14(11):1375. doi: 10.3390/life14111375 (PMC11595905; doi:10.3390/life14111375)
Supplement: Supplementary file 1 [file life-14-01375-s001.zip › life-3238287-supplementary.pdf]

Ammonia Sample 3 Methanol B

C:\GCMSsolution\Data\Project1\Ms Mathe\SAMPLE 3_UNKNOWN_20221130_3.qgd

Chromatogram (All TIC)]

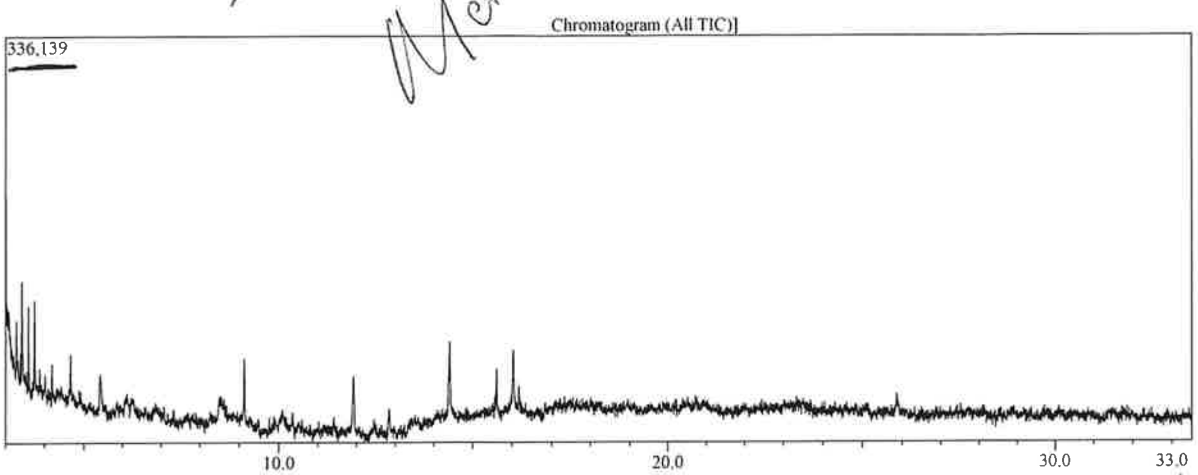

Chromatogram (Zoom)

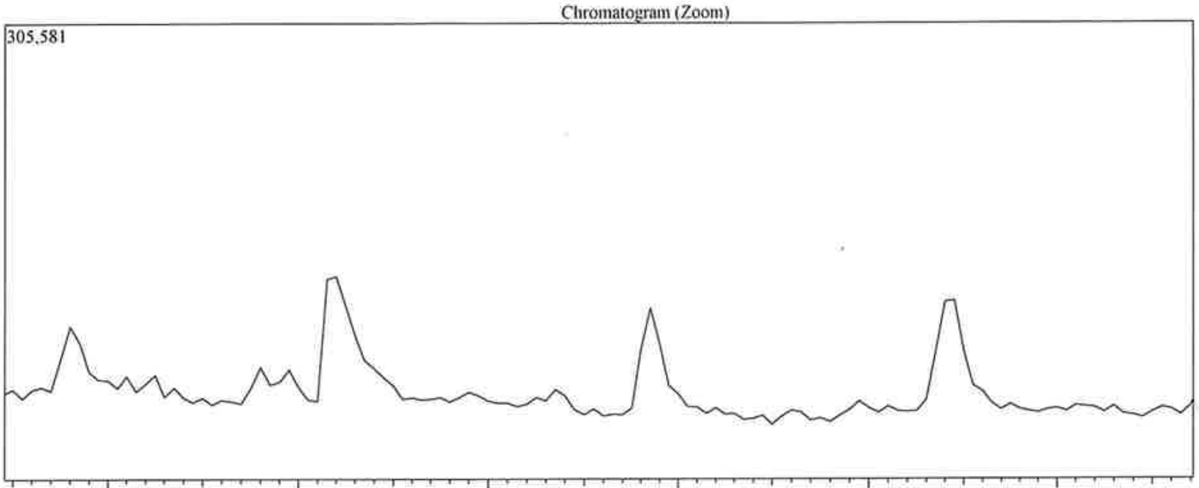

Spectrum

Line#:1 R.Time:3.280(Scan#:57)
MassPeaks:598
RawMode:Single 3.280(57) BasePeak:18.10(33884)
BG Mode:None Group 1 - Event 1

x100 10,080

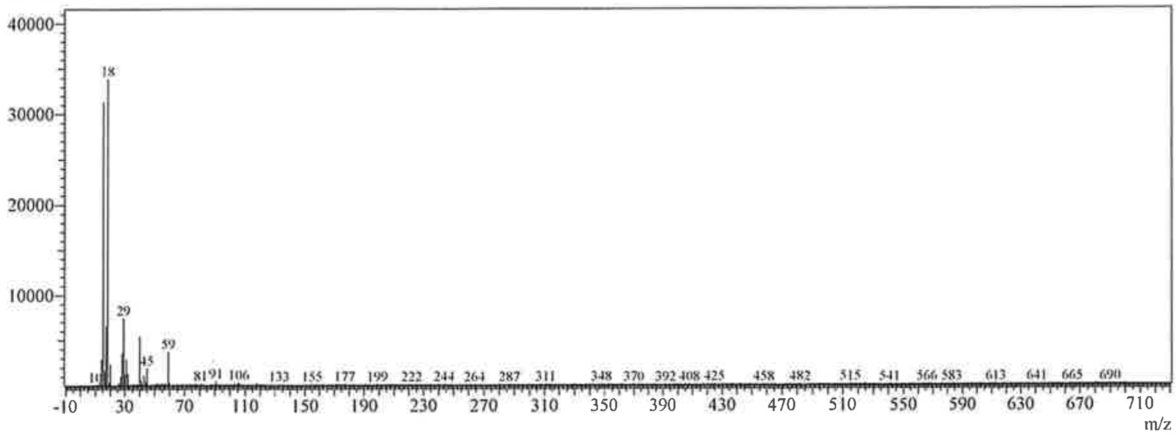

Similarity Search Results

Report View Compound Info Process Help

Hit	Similar	Regi	Compound Name	Mol Wt	Formula	Library
1	69	☑	Glycidol $$ Oxiranemethanol $$ 1-Propanol, 2,	74	C3H6O2	NIST08.LIB
2	69	☐	Ethanedioic acid, dimethyl ester $$ Oxalic acid	118	C4H6O4	NIST08.LIB
3	67	☐	Carbonic acid, dimethyl ester $$ Dimethyl carb	90	C3H6O3	NIST08.LIB
4	67	☐	Ethene, methoxy- $$ Ether, methyl vinyl $$ Me	58	C3H6O	NIST08.LIB
5	66	☐	Acetonitrile, hydroxy- $$ Glycolonitrile $$ Cyan	57	C2H3NO	NIST08.LIB
6	66	☐	Ethylene oxide $$ Oxirane $$ Dihydrooxirene	44	C2H4O	NIST08.LIB
7	65	☐	Ethane, methoxy- $$ Ether, ethyl methyl $$ Et	60	C3H8O	NIST08.LIB
8	65	☐	Butanoic acid, 4-(2,4,5-trichlorophenoxy)-, met	296	C11H11Cl3O3	NIST08.LIB
9	65	☐	Hydrazine, 1,1-dimethyl- $$ aS-Dimethylhydrazi	60	C2H8N2	NIST08.LIB
10	64	☐	Acetaldoxime $$ (E)-CH3CH=NOH $$ Acetald	59	C2H5NO	NIST08.LIB
11	64	☐	2-Propanone, 1,3-dihydroxy- $$ Dihydroxyacet	90	C3H6O3	NIST08.LIB

Target:

Base Peak: 18/ 10,000

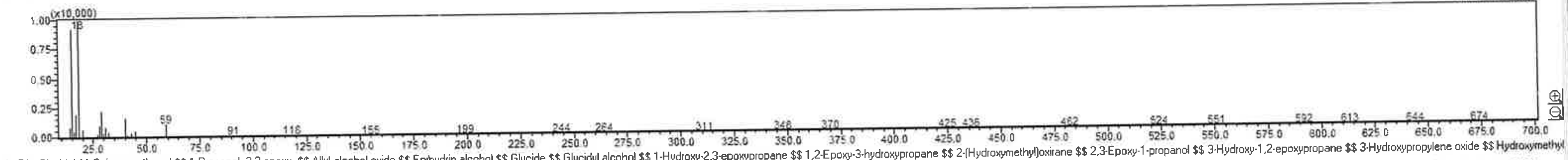

1 : 74 : Glycidol $$ Oxiranemethanol $$ 1-Propanol, 2,3-epoxy- $$ Allyl alcohol oxide $$ Epihydrin alcohol $$ Glycide $$ Glycidyl alcohol $$ 1-Hydroxy-2,3-epoxypropane $$ 1,2-Epoxy-3-hydroxypropane $$ 2-(Hydroxymethyl)oxirane $$ 2,3-Epoxy-1-propanol $$ 3-Hydroxy-1,2-epoxypropane $$ 3-Hydroxypropylene oxide $$ Hydroxymethyl

Base Peak: 44/ 10,000

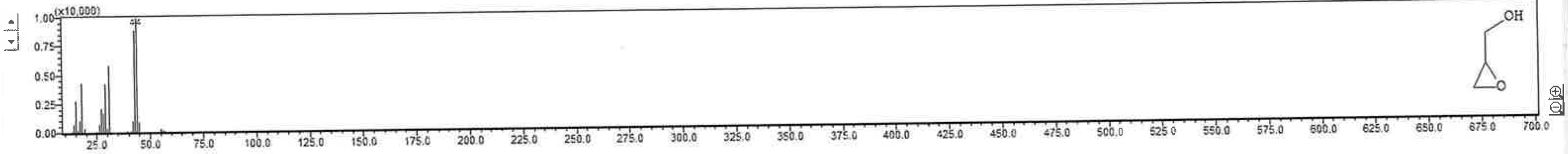

CAS#: 556 - 52 - 5 Mol Wt: 74 Serial#: 375

Cmpd Name: Glycidol $$ Oxiranemethanol $$ 1-Propanol, 2,3-epoxy- $$ Allyl alcohol oxide $$ Epihydrin alcohol $$ Glycide $$ Glycidyl alcohol $$ 1-Hydroxy-2,3-epoxypropane $$ 1,2-Epoxy-3-hydroxypropane $$ 2-(Hydroxymethyl)oxirane $$ 2,3-Epoxy-1-propanol $$ 3-Hydroxy-1,2-epoxypropane $$ 3-Hydroxypropylene oxide $$ Hydr

Formula: C3H6O2 Class Flag: No Class Flags

Ret.Index: 653

11:37 AM 2022/12/06

C:\GCMSsolution\Data\Project1\Ms Mathe\SAMPLE 3_UNKNOWN _20221130_3.qgd

Chromatogram (All TIC)]

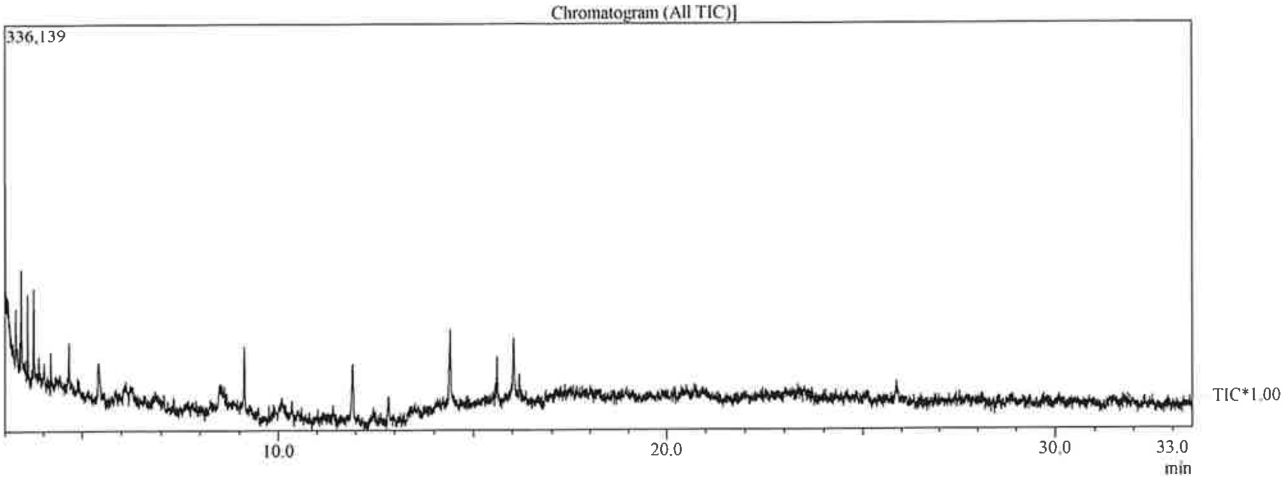

Chromatogram (Zoom)

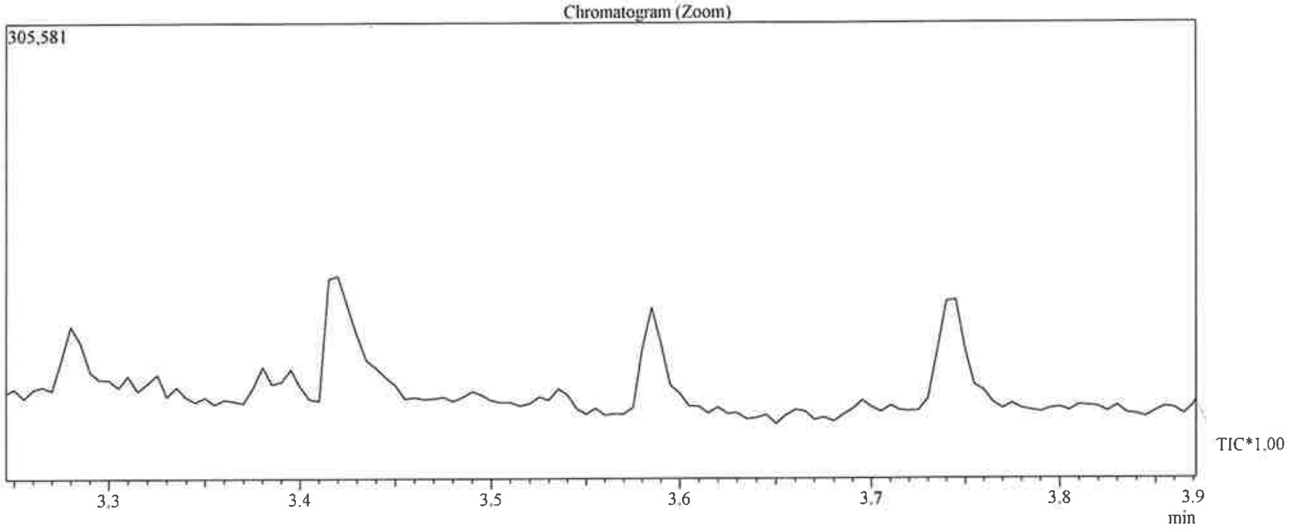

Spectrum

Line#:1 R.Time:3.420(Scan#:85)
MassPeaks:579
RawMode:Single 3.420(85) BasePeak:18.10(31369)
BG Mode:None Group 1 - Event 1

9,332

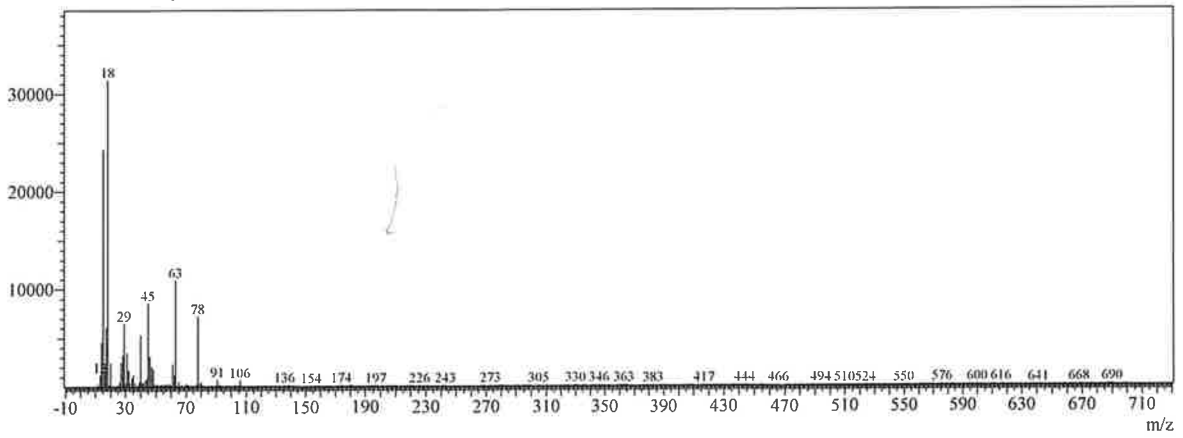

Similarity Search Results

Report View Compound Info Process Help

Hit	Similar	Regi	Compound Name	Mol Wt	Formula	Library
1	70	☑	Dimethyl Sulfoxide $$ DMSO $$ Methane, sulf	78	C2H6OS	NIST08.LIB
2	66	☐	Carbonic acid, dimethyl ester $$ Dimethyl carb	90	C3H6O3	NIST08.LIB
3	66	☐	Dimethoxyamine $$ N-Methoxy-O-methylhydro	77	C2H7NO2	NIST08.LIB
4	64	☐	Peroxide, diethyl $$ Ethyl peroxide $$ Diethyl	90	C4H10O2	NIST08.LIB
5	63	☐	Glycidol $$ Oxiranemethanol $$ 1-Propanol, 2,	74	C3H6O2	NIST08.LIB
6	63	☐	Acetyl chloride $$ Ethanoyl chloride $$ Acetic	78	C2H3ClO	NIST08.LIB
7	63	☐	Methane, chloromethoxy- $$ Ether, chloromet	80	C2H5ClO	NIST08.LIB
8	62	☐	Fluoroacetic acid $$ Acetic acid, fluoro- $$ Cy	78	C2H3FO2	NIST08.LIB
9	62	☐	2-Butanol, 3-chloro-, (R*,R*)- $$ 2-Butanol, 3-c	108	C4H9ClO	NIST08.LIB
10	62	☐	Carbonic acid, ethyl-, methyl ester $$ Ethoxya	104	C4H8O3	NIST08.LIB
11	62	☐	2-Methanesulfonylethanol $$ 2-Hydroxyethylm	124	C3H8O3S	NIST08.LIB

Target:

Base Peak: 18/ 10,000

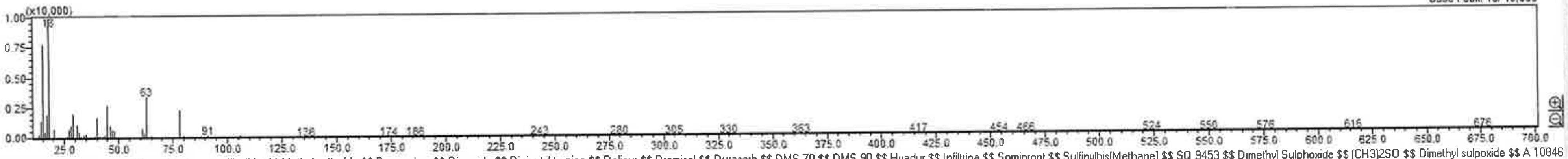

1 : 78 : Dimethyl Sulfoxide $$ DMSO $$ Methane, sulfinylbis- $$ Methyl sulfoxide $$ Demsodrox $$ Dimexide $$ Dipirartril-tropico $$ Dolicur $$ Dromisol $$ Durasorb $$ DMS 70 $$ DMS 90 $$ Hyadur $$ Infiltrina $$ Somipront $$ Sulfinylbis[Methane] $$ SQ 9453 $$ Dimethyl Sulphoxide $$ (CH3)2SO $$ Dimethyl sulpoxide $$ A 10846

Base Peak: 63/ 10,000

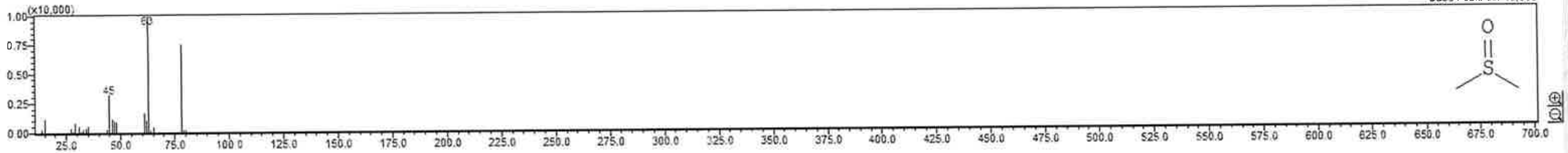

CAS#: 67 - 68 - 5 Mol Wt: 78 Serial#: 468

Cmpd Name: Dimethyl Sulfoxide $$ DMSO $$ Methane, sulfinylbis- $$ Methyl sulfoxide $$ Demsodrox $$ Dimexide $$ Dipirartril-tropico $$ Dolicur $$ Dromisol $$ Durasorb $$ DMS 70 $$ DMS 90 $$ Hyadur $$ Infiltrina $$ Somipront $$ Sulfinylbis[Methane] $$ SQ 9453 $$ Dimethyl Sulphoxide $$ (CH3)2SO $$ Dimethyl sulpoxide $$

Formula: C2H6OS Class Flag: No Class Flags.

Ret.Index: 691

11:38 AM 2022/12/06

C:\GCMSsolution\Data\Project1\Ms Mathe\SAMPLE 3_UNKNOWN _20221130_3.qgd

Chromatogram (All TIC)]

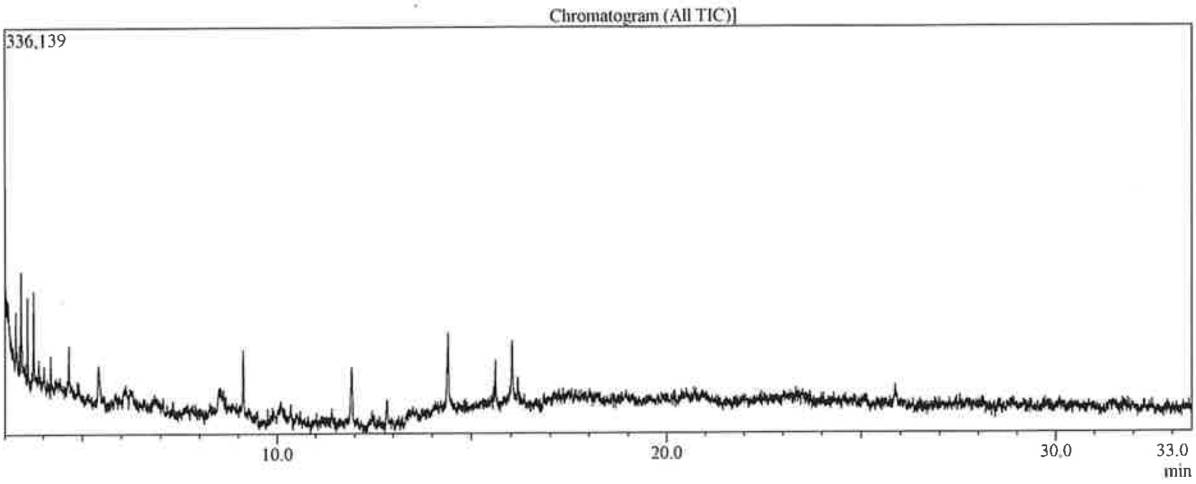

Chromatogram (Zoom)

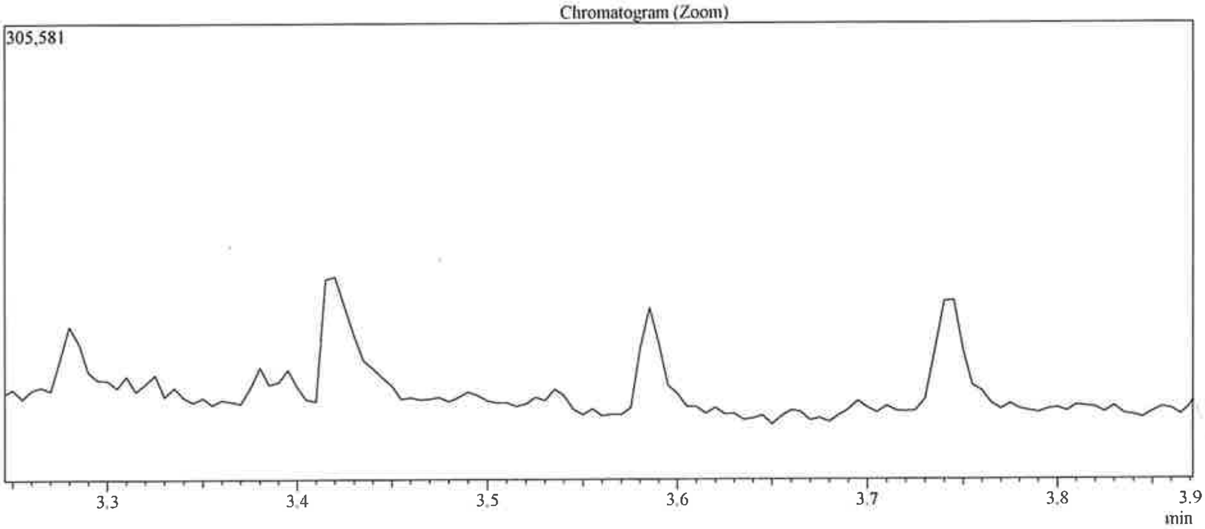

Spectrum

Line#:1 R.Time:3.585(Scan#:118)
MassPeaks:593
RawMode:Single 3.585(118) BasePeak:18.10(25916)
BG Mode:None Group 1 - Event 1

7,710

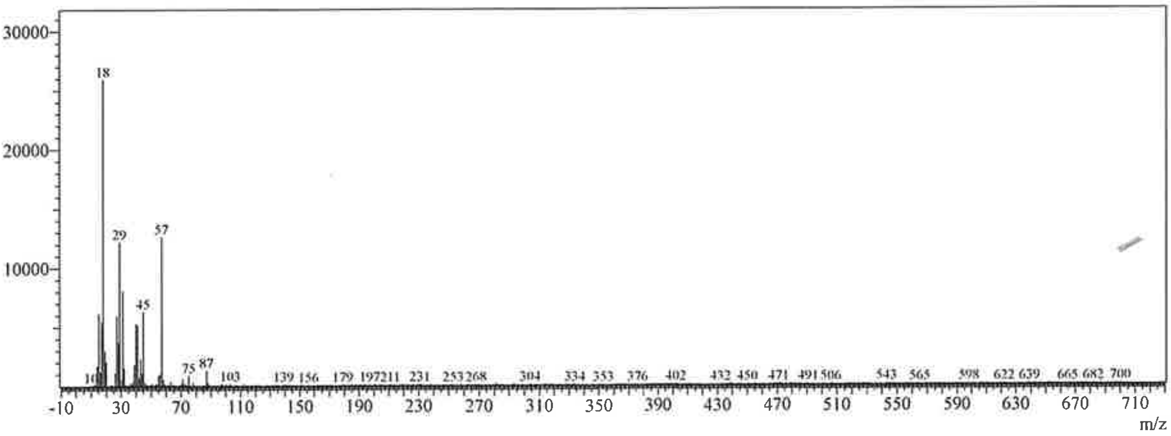

Similarity Search Results

Report View Compound Info Process Help

Hit	Similar	Regi	Compound Name	Mol Wt	Formula	Library
1	76	☑	Propanoic acid, 2-hydroxyethyl ester $$ 2-Hydr	118	C5H10O3	NIST08.LIB
2	75	☐	Glycidol $$ Oxiranemethanol $$ 1-Propanol, 2,	74	C3H6O2	NIST08.LIB
3	74	☐	Formic acid, 1-methylpropyl ester $$ Formic aci	102	C5H10O2	NIST08.LIB
4	74	☐	2-Hydroxy-3-pentanone $$ 3-Pentanone, 2-hy	102	C5H10O2	NIST08.LIB
5	74	☐	Propanoic acid, 2-methylpropyl ester $$ Propio	130	C7H14O2	NIST08.LIB
6	74	☐	Oxirane, (ethoxymethyl)- $$ Ethyl glycidyl ether	102	C5H10O2	NIST08.LIB
7	74	☐	Butane, 2,2'-[methylenebis(oxy)]bis- $$ Methan	160	C9H20O2	NIST08.LIB
8	73	☐	1,3-Propanediol, 2-(hydroxymethyl)-2-nitro- $$ I	151	C4H9NO5	NIST08.LIB
9	73	☐	Butyl glyoxylate $$ Acetic acid, oxo-, butyl est	130	C6H10O3	NIST08.LIB
10	73	☐	1,3-Butanediol $$ .beta.-Butylene glycol $$ M	90	C4H10O2	NIST08.LIB
11	73	☐	1-Hydroxy-2-butanone $$ 2-Butanone, 1-hydro	88	C4H8O2	NIST08.LIB

Target:

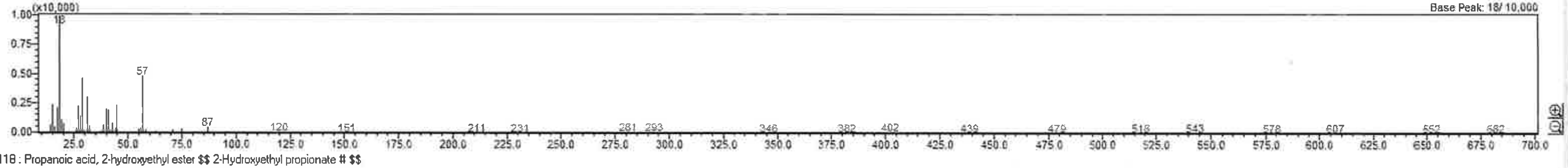

1 : 118 : Propanoic acid, 2-hydroxyethyl ester $$ 2-Hydroxyethyl propionate # $$

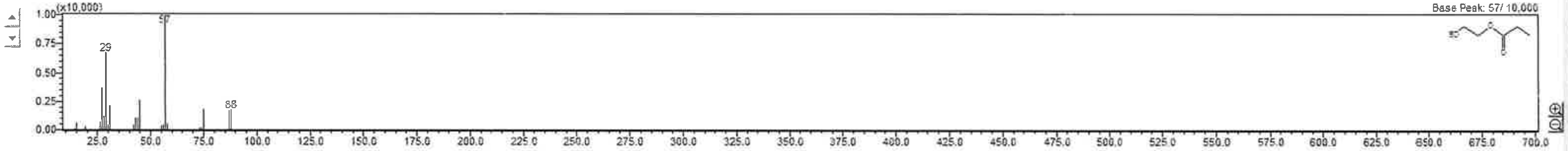

CAS#: 24567 - 27 - 9 Mol Wt: 118 Serial#: 4913

Cmpd Name: Propanoic acid, 2-hydroxyethyl ester $$ 2-Hydroxyethyl propionate # $$

Formula: C5H10O3 Class Flag: No Class Flags.

Ret.Index: 928

11:39 AM
2022/12/06

C:\GCMSsolution\Data\Project1\Ms Mathe\SAMPLE 3_UNKNOWN _20221130_3.qgd

Chromatogram (All TIC)]

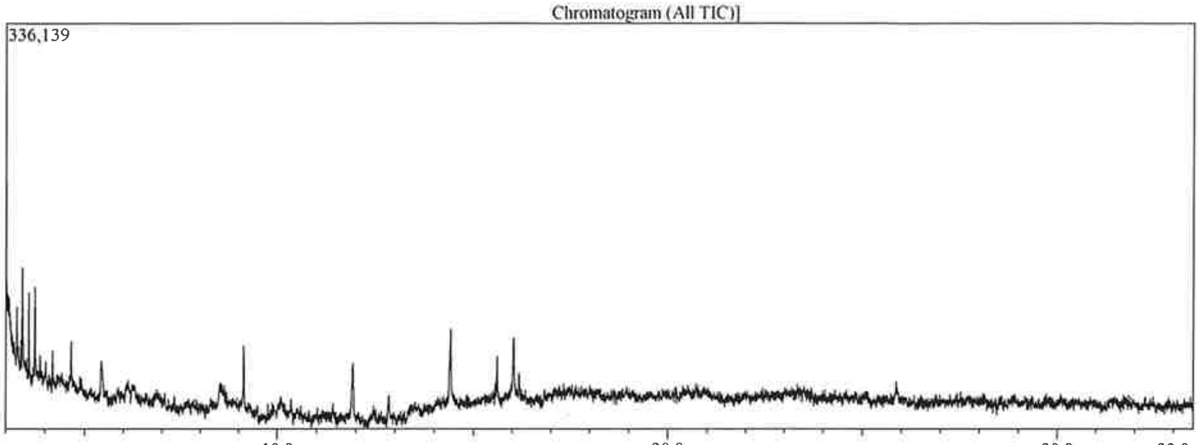

Chromatogram (Zoom)

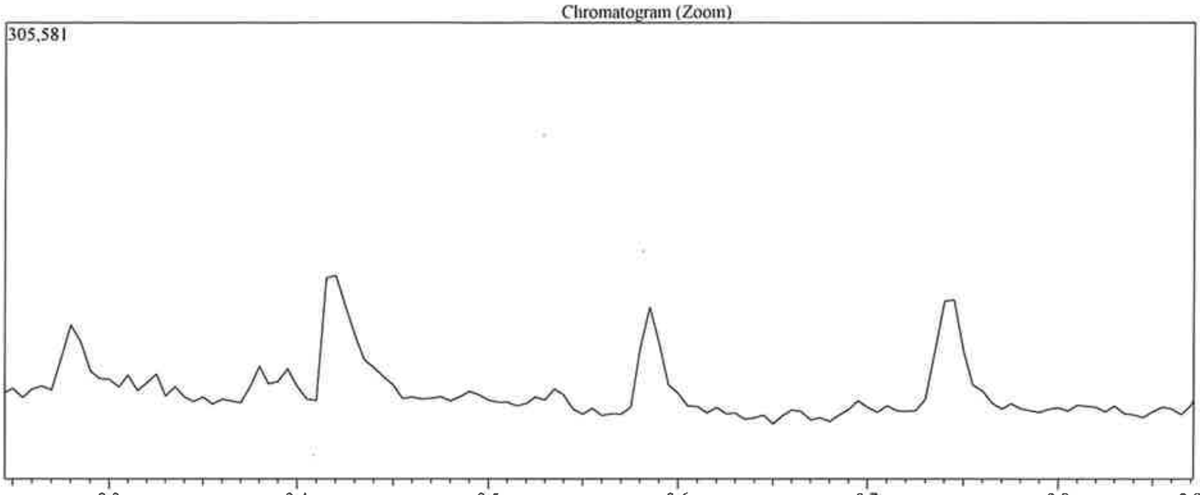

Spectrum

Line#:1 R.Time:3.745(Scan#:150)
MassPeaks:597
RawMode:Single 3.745(150) BasePeak:15.15(48979)
BG Mode:None Group 1 - Event 1

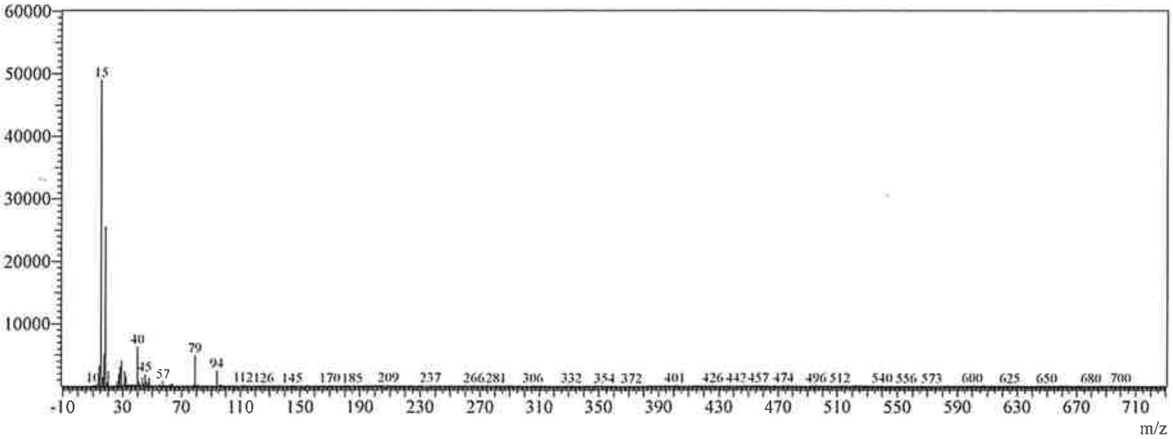

Similarity Search Results

Report View Compound Info Process Help

Hit	Similar	Regi	Compound Name	Mol Wt	Formula	Library
1	70	☑	Dimethyl sulfone $$ Methane, sulfonylbis- $$	94	C2H6O2S	NIST08.LIB
2	66	☐	Glycidol $$ Oxiranemethanol $$ 1-Propanol, 2,	74	C3H6O2	NIST08.LIB
3	64	☐	Ethylene oxide $$ Oxirane $$ Dihydrooxirene	44	C2H4O	NIST08.LIB
4	63	☐	anti-2-Acetoxyacetaldoxime $$ (2E)-2-(Hydroxy	117	C4H7NO3	NIST08.LIB
5	63	☐	Aziridine, 1-methyl- $$ N-Methylaziridine $$ N-	57	C3H7N	NIST08.LIB
6	62	☐	Acetyl bromide $$ CH3COBr $$ UN 1716 $$	122	C2H3BrO	NIST08.LIB
7	62	☐	Acetonitrile, hydroxy- $$ Glycolonitrile $$ Cyan	57	C2H3NO	NIST08.LIB
8	62	☐	Methane, nitro- $$ Nitromethane $$ Nitrocarbo	61	CH3NO2	NIST08.LIB
9	62	☐	Carbonic acid, dimethyl ester $$ Dimethyl carb	90	C3H6O3	NIST08.LIB
10	62	☐	Dimethylfluoroamine $$ N-Fluoro-N-methylmeth	63	C2H6FN	NIST08.LIB
11	62	☐	2-Hydroxyimino-3-oxo-butanoic acid, methyl es	145	C5H7NO4	NIST08.LIB

Target:

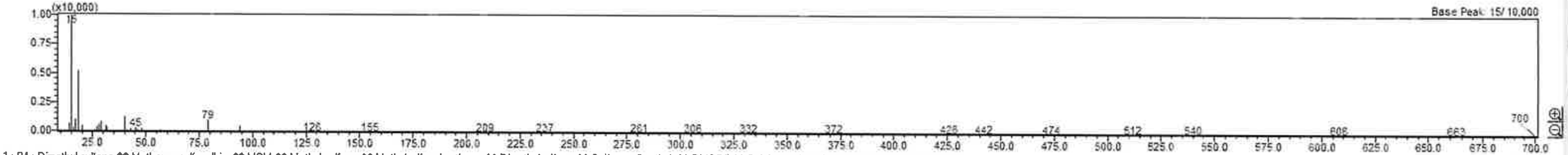

1 : 94 : Dimethyl sulfone $$ Methane, sulfonylbis- $$ MSM $$ Methyl sulfone $$ Methylsulfonylmethane $$ Dimethylsulfone $$ Sulfone, dimethyl- $$ DMSO2 $$ Sulphonylbismethane $$ Dimethyl sulphone $$

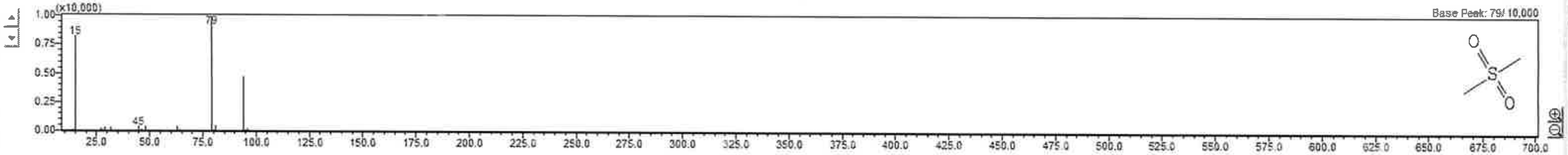

CAS#: 67 - 71 - 0 Mol Wt: 94 Serial#: 1234

Cmpd Name: Dimethyl sulfone $$ Methane, sulfonylbis- $$ MSM $$ Methyl sulfone $$ Methylsulfonylmethane $$ Dimethylsulfone $$ Sulfone, dimethyl- $$ DMSO2 $$ Sulphonylbismethane $$ Dimethyl sulphone $$

Formula: C2H6O2S Class Flag: No Class Flags.

Ret.Index: 727

11:39 AM 2022/12/06

C:\GCMSsolution\Data\Project1\Ms Mathe\SAMPLE 3_UNKNOWN _20221130_3.qgd

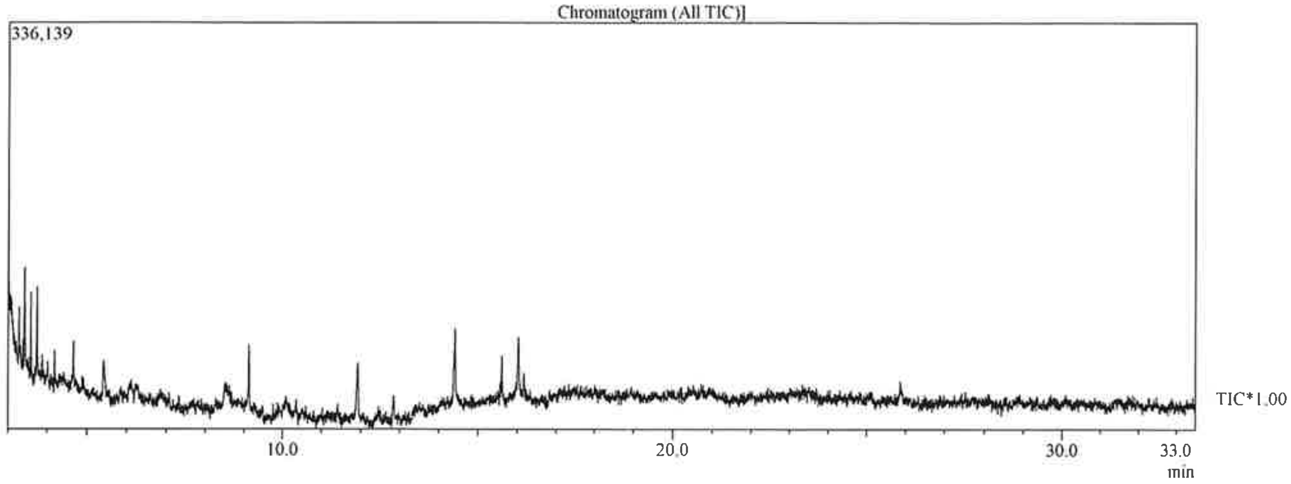

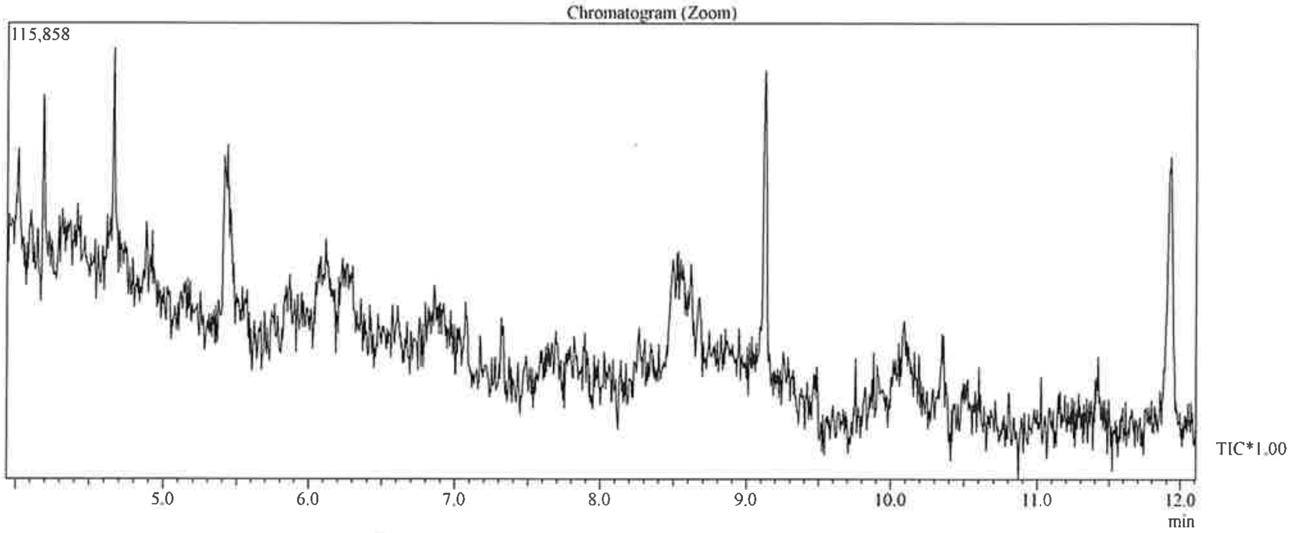

1

Spectrum

Line#:1 R.Time:4.015(Scan#:204)
MassPeaks:583
RawMode:Single 4.015(204) BasePeak:18.10(24991)
BG Mode:None Group 1 - Event 1

7,435

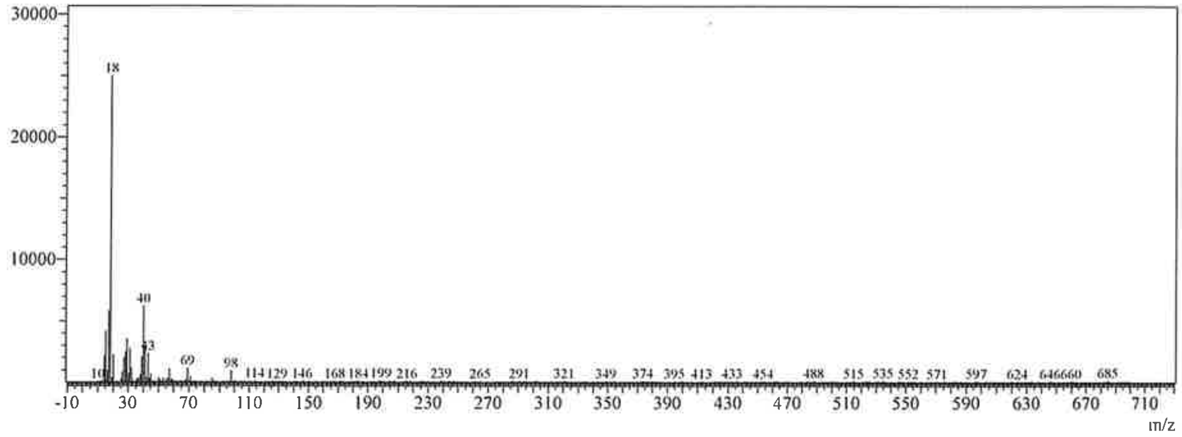

Similarity Search Results

Report View Compound Info Process Help

Hit	Similar	Regi	Compound Name	Mol Wt	Formula	Library
1	69	☑	Glycidol $$ Oxiranemethanol $$ 1-Propanol, 2	74	C3H6O2	NIST08.LIB
2	69	☐	Acetonitrile, hydroxy- $$ Glycolonitrile $$ Cyan	57	C2H3NO	NIST08.LIB
3	68	☐	1-Hydroxy-2-pentanone $$ 2-Pentanone, 1-hy	102	C5H10O2	NIST08.LIB
4	68	☐	Glycyl-D-asparagine	189	C6H11N3O4	NIST08.LIB
5	66	☐	2-Butenedioic acid, 2-methyl-, (Z)- $$ Citraconi	130	C5H6O4	NIST08.LIB
6	66	☐	2-Propanone, 1,3-dihydroxy- $$ Dihydroxyacet	90	C3H6O3	NIST08.LIB
7	66	☐	1-Propanol, 2-amino-, (+/-)- $$ 1-Propanol, 2-	75	C3H9NO	NIST08.LIB
8	66	☐	Ethanol, 2-(2-propynyloxy)- $$ Ethylene glycol	100	C5H8O2	NIST08.LIB
9	65	☐	2-Nitro-1-propanol $$ 2-Nitropropanol-1 $$ 1-P	105	C3H7NO3	NIST08.LIB
10	65	☐	Azetidine, 1,3-dimethyl- $$ 1,3-Dimethylazetidi	85	C5H11N	NIST08.LIB
11	65	☐	Propane, 2-methoxy- $$ Ether, isopropyl methy	74	C4H10O	NIST08.LIB

Target:

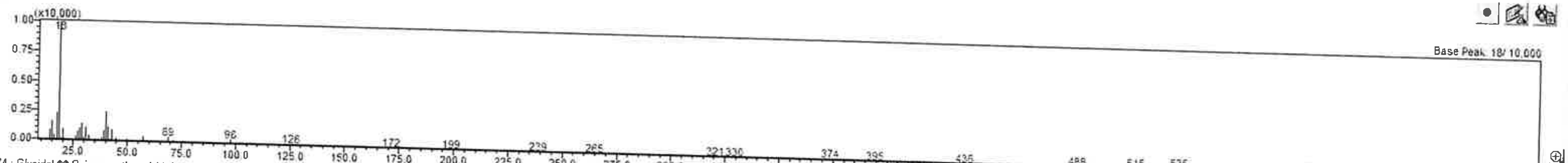

1 : 74 : Glycidol $$ Oxiranemethanol $$ 1-Propanol, 2,3-epoxy- $$ Allyl alcohol oxide $$ Epihydrin alcohol $$ Glycide $$ Glycidyl alcohol $$ 1-Hydroxy-2,3-epoxypropane $$ 1,2-Epoxy-3-hydroxypropane $$ 2-(Hydroxymethyl)oxirane $$ 2,3-Epoxy-1-propanol $$ 3-Hydroxy-1,2-epoxypropane $$ 3-Hydroxypropylene oxide $$ Hydroxymethyl

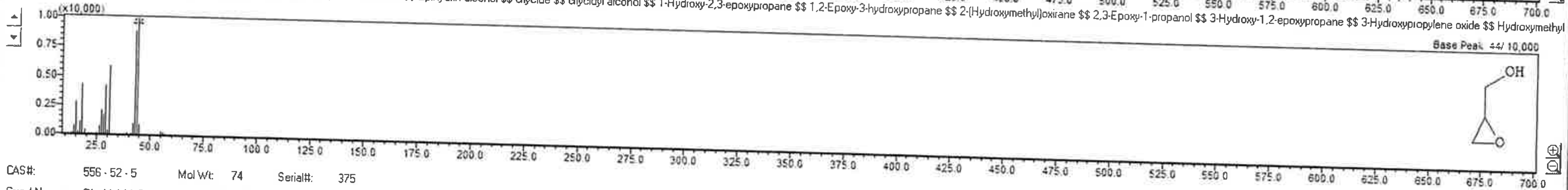

CAS#: 556-52-5 Mol Wt: 74 Serial#: 375

Cmpd Name: Glycidol $$ Oxiranemethanol $$ 1-Propanol, 2,3-epoxy- $$ Allyl alcohol oxide $$ Epihydrin alcohol $$ Glycide $$ Glycidyl alcohol $$ 1-Hydroxy-2,3-epoxypropane $$ 1,2-Epoxy-3-hydroxypropane $$ 2-(Hydroxymethyl)oxirane $$ 2,3-Epoxy-1-propanol $$ 3-Hydroxy-1,2-epoxypropane $$ 3-Hydroxypropylene oxide $$ Hydr

Formula: C3H6O2 Class Flag: No Class Flags

Ret.Index: 653

11:40 AM
2022/12/06

C:\GCMSsolution\Data\Project1\Ms Mathe\SAMPLE 3_UNKNOWN _20221130_3.qgd

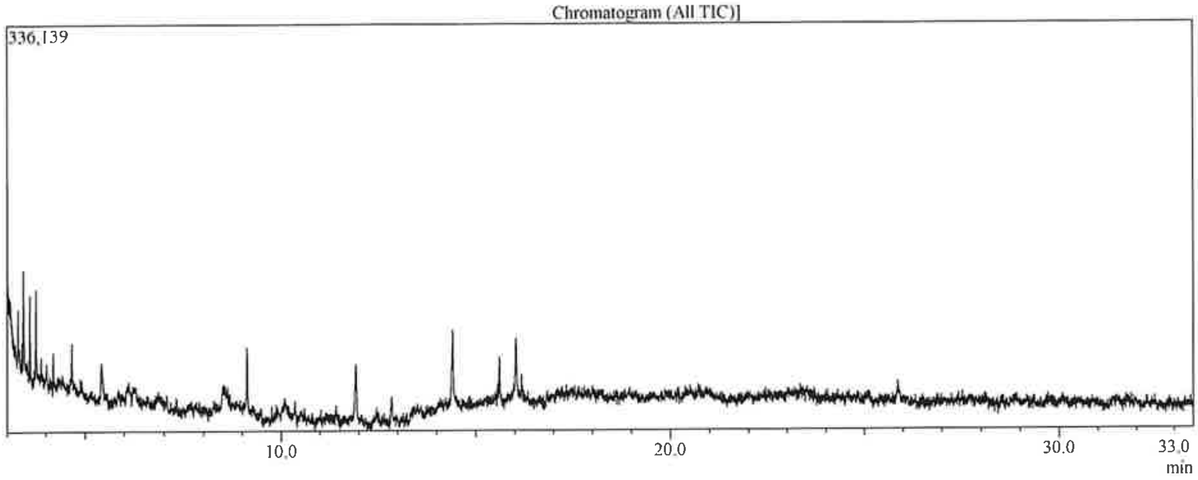

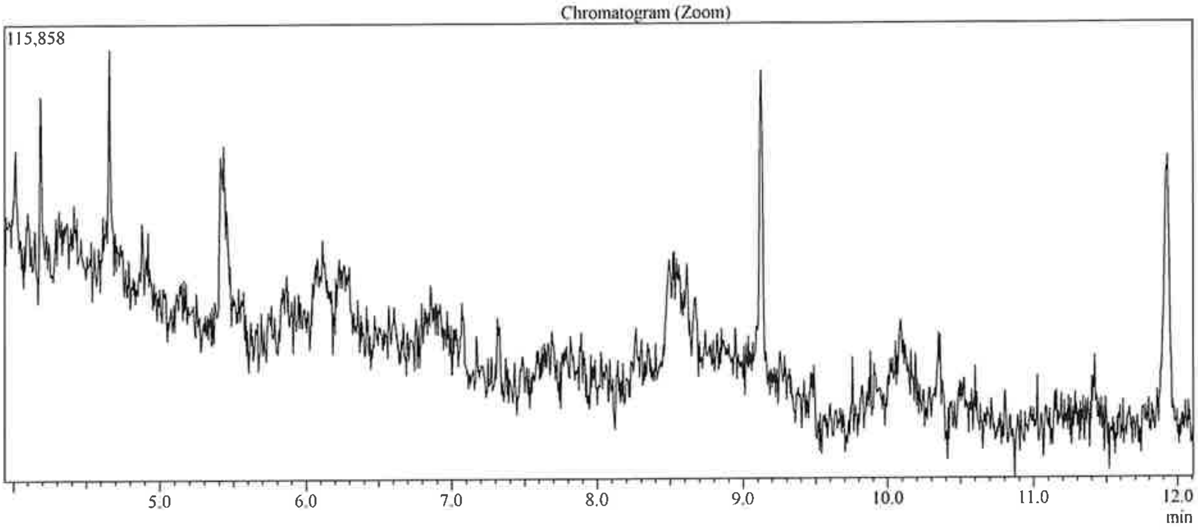

Spectrum

② 6,703

Line#:1 R.Time:4.190(Scan#:239)
MassPeaks:579
RawMode:Single 4.190(239) BasePeak:18.10(22530)
BG Mode:None Group 1 - Event 1

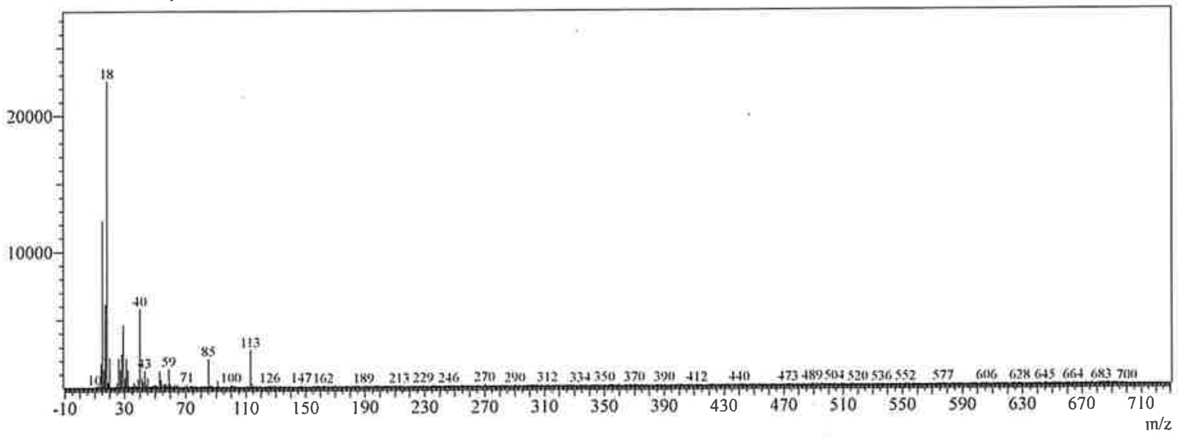

Similarity Search Results

Report View Compound Info Process Help

Hit	Similar	Regi	Compound Name	Mol Wt	Formula	Library
1	69	☑	Glycidol $$ Oxiranemethanol $$ 1-Propanol, 2	74	C3H6O2	NIST08.LIB
2	67	☐	Glycyl-D-asparagine	189	C6H11N3O4	NIST08.LIB
3	67	☐	Acetonitrile, hydroxy- $$ Glycolonitrile $$ Cyan	57	C2H3NO	NIST08.LIB
4	65	☐	Aziridine, 1-methyl- $$ N-Methylaziridine $$ N-	57	C3H7N	NIST08.LIB
5	65	☐	DL-Arabinose $$ Pentopyranose # $$	150	C5H10O5	NIST08.LIB
6	64	☐	2-Hydroxyimino-3-oxo-butanoic acid, methyl es	145	C5H7NO4	NIST08.LIB
7	64	☐	2-Propanone, 1,3-dihydroxy- $$ Dihydroxyacet	90	C3H6O3	NIST08.LIB
8	64	☐	Acetaldoxime $$ (E)-CH3CH=NOH $$ Acetald	59	C2H5NO	NIST08.LIB
9	63	☐	N-Ethoxy-2-carbaminoaziridine $$ 1-Ethoxy-2-a	130	C5H10N2O2	NIST08.LIB
10	63	☐	Ethene, methoxy- $$ Ether, methyl vinyl $$ Me	58	C3H6O	NIST08.LIB
11	63	☐	1,3-Dihydroxyacetone dimer	180	C6H12O6	NIST08.LIB

Target:

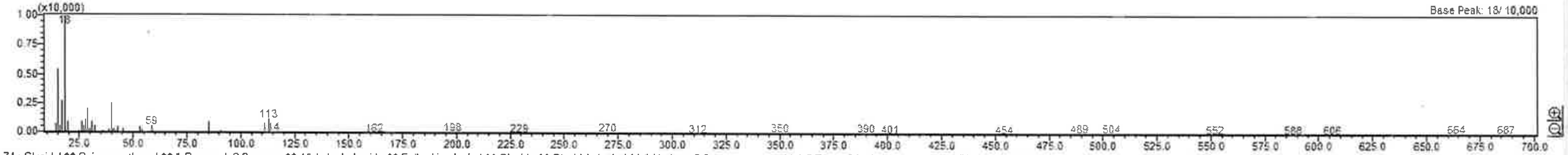

1 : 74 : Glycidol $$ Oxiranemethanol $$ 1-Propanol, 2,3-epoxy- $$ Allyl alcohol oxide $$ Epihydrin alcohol $$ Glycide $$ Glycidyl alcohol $$ 1-Hydroxy-2,3-epoxypropane $$ 1,2-Epoxy-3-hydroxypropane $$ 2-(Hydroxymethyl)oxirane $$ 2,3-Epoxy-1-propanol $$ 3-Hydroxy-1,2-epoxypropane $$ 3-Hydroxypropylene oxide $$ Hydroxymethyl

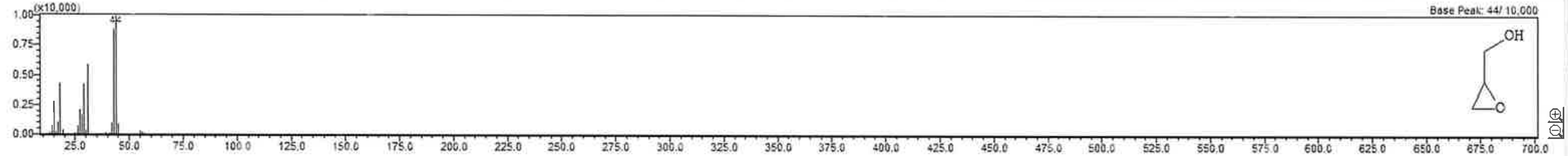

CAS#: 556 - 52 - 5 Mol Wt: 74 Serial#: 375

Cmpd Name: Glycidol $$ Oxiranemethanol $$ 1-Propanol, 2,3-epoxy- $$ Allyl alcohol oxide $$ Epihydrin alcohol $$ Glycide $$ Glycidyl alcohol $$ 1-Hydroxy-2,3-epoxypropane $$ 1,2-Epoxy-3-hydroxypropane $$ 2-(Hydroxymethyl)oxirane $$ 2,3-Epoxy-1-propanol $$ 3-Hydroxy-1,2-epoxypropane $$ 3-Hydroxypropylene oxide $$ Hydrc

Formula: C3H6O2 Class Flag No Class Flags

Ret.Index: 653

11:41 AM 2022/12/06

C:\GCMSsolution\Data\Project1\Ms Mathe\SAMPLE 3_UNKNOWN _20221130_3.qgd

Chromatogram (All TIC)]

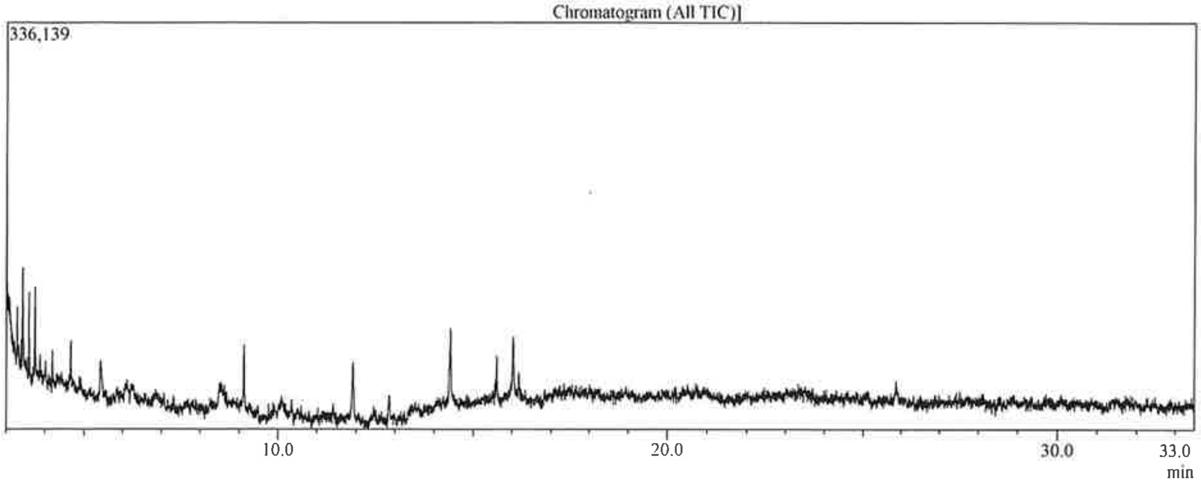

Chromatogram (Zoom)

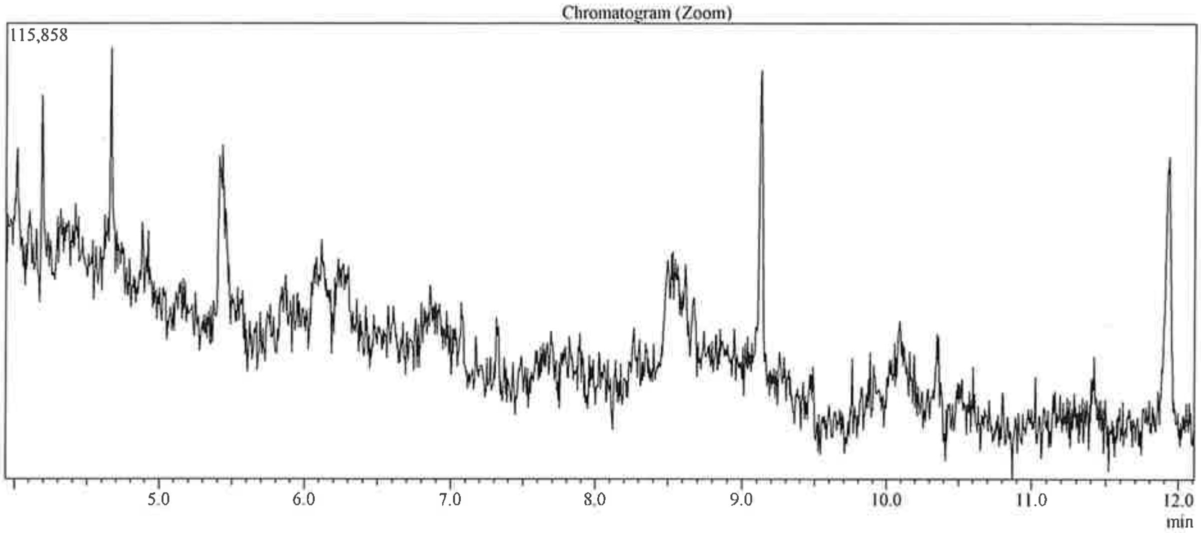

Spectrum

③ 5.829

Line#:1 R.Time:4.660(Scan#:333)
MassPeaks:609
RawMode:Single 4.660(333) BasePeak:18.10(19593)
BG Mode:None Group 1 - Event 1

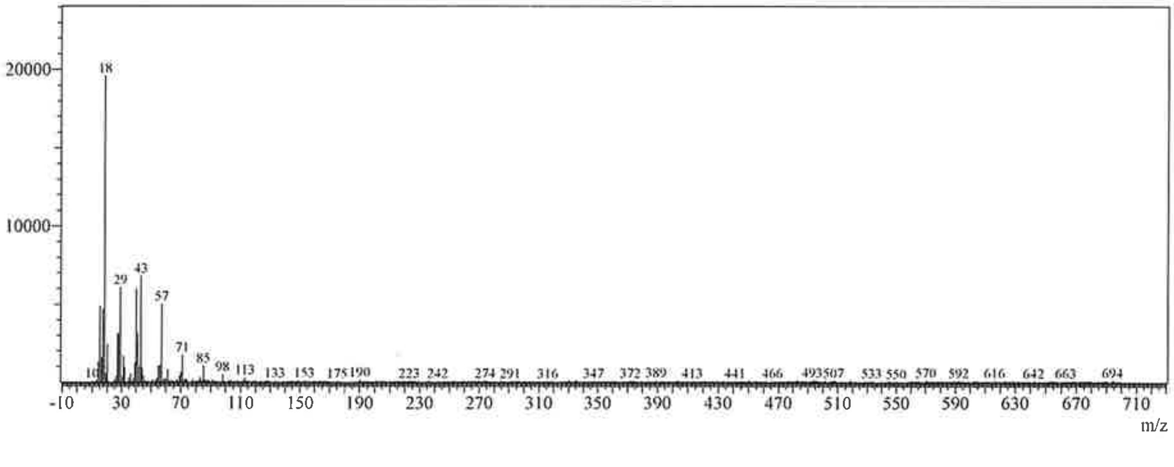

Similarity Search Results

Report View Compound Info Process Help

Hit	Similar	Regi	Compound Name	Mol Wt	Formula	Library
1	73	☑	Glycidol $$ Oxiranemethanol $$ 1-Propanol, 2	74	C3H6O2	NIST08.LIB
2	72	☐	DL-Arabinose $$ Pentopyranose # $$	150	C5H10O5	NIST08.LIB
3	71	☐	Glycyl-D-asparagine	189	C6H11N3O4	NIST08.LIB
4	71	☐	3,3-Dimethyl-2-pentanone $$ 3,3-Dimethyl-2-p	114	C7H14O	NIST08.LIB
5	71	☐	1-Hydroxy-2-pentanone $$ 2-Pentanone, 1-hy	102	C5H10O2	NIST08.LIB
6	70	☐	Hexane, 1-(ethenyloxy)- $$ Ether, hexyl vinyl $	128	C8H16O	NIST08.LIB
7	70	☐	Propane, 2-methoxy- $$ Ether, isopropyl methy	74	C4H10O	NIST08.LIB
8	70	☐	(2S,3S)-(-)-3-Propyloxiranemethanol $$ (3-Prop	116	C6H12O2	NIST08.LIB
9	70	☐	3-Methyl-3-oxetanemethanol $$ (3-Methyl-3-ox	102	C5H10O2	NIST08.LIB
10	70	☐	Peroxide, bis(1-methylethyl) $$ Isopropyl peroxi	118	C6H14O2	NIST08.LIB
11	70	☐	Hydroperoxide, 1-methylhexyl $$ n-C5H11CH(	132	C7H16O2	NIST08.LIB

Target:

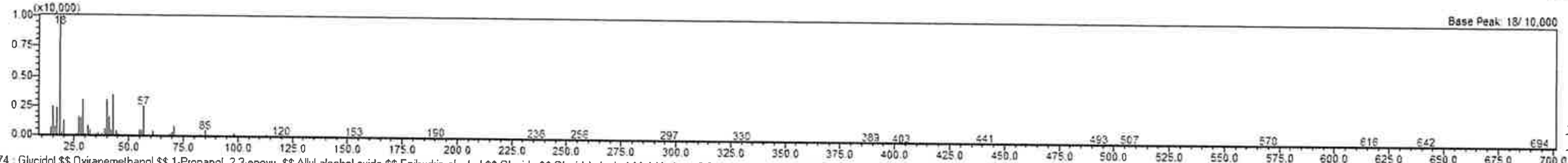

1 : 74 : Glycidol $$ Oxiranemethanol $$ 1-Propanol, 2,3-epoxy- $$ Allyl alcohol oxide $$ Epihydrin alcohol $$ Glycide $$ Glycidyl alcohol $$ 1-Hydroxy-2,3-epoxypropane $$ 1,2-Epoxy-3-hydroxypropane $$ 2-(Hydroxymethyl)oxirane $$ 2,3-Epoxy-1-propanol $$ 3-Hydroxy-1,2-epoxypropane $$ 3-Hydroxypropylene oxide $$ Hydroxymethyl

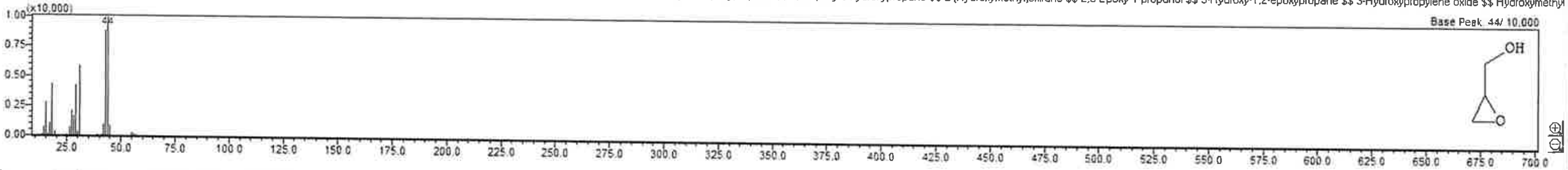

CAS#: 556-52-5 Mol Wt: 74 Serial#: 375

Cmpd Name: Glycidol $$ Oxiranemethanol $$ 1-Propanol, 2,3-epoxy- $$ Allyl alcohol oxide $$ Epihydrin alcohol $$ Glycide $$ Glycidyl alcohol $$ 1-Hydroxy-2,3-epoxypropane $$ 1,2-Epoxy-3-hydroxypropane $$ 2-(Hydroxymethyl)oxirane $$ 2,3-Epoxy-1-propanol $$ 3-Hydroxy-1,2-epoxypropane $$ 3-Hydroxypropylene oxide $$ Hydr

Formula: C3H6O2 Class Flag: No Class Flags

Ret.Index: 653

11:41 AM
2022/12/06

C:\GCMSsolution\Data\Project1\Ms Mathe\SAMPLE 3_UNKNOWN _20221130_3.qgd

Chromatogram (All TIC)]

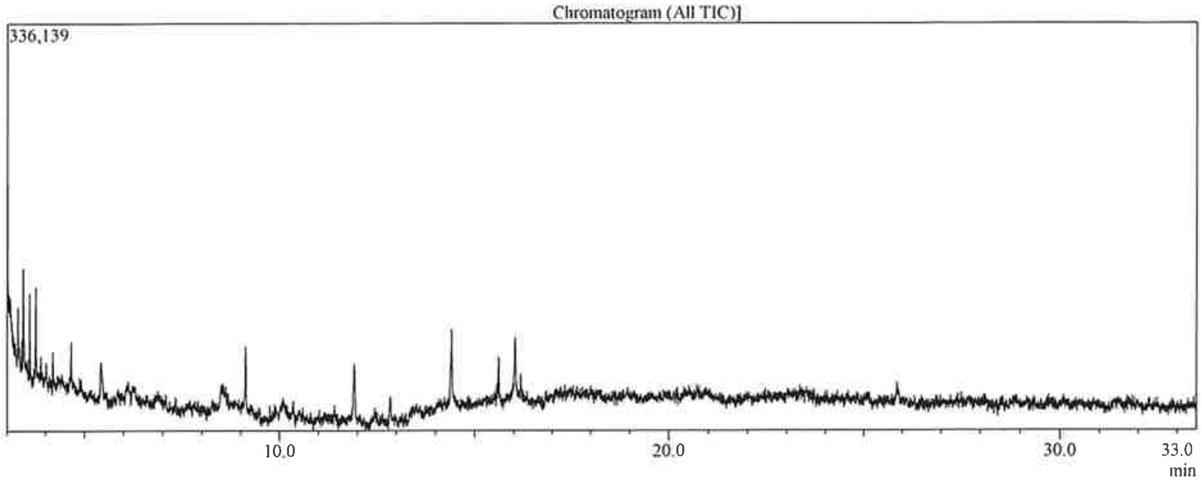

Chromatogram (Zoom)

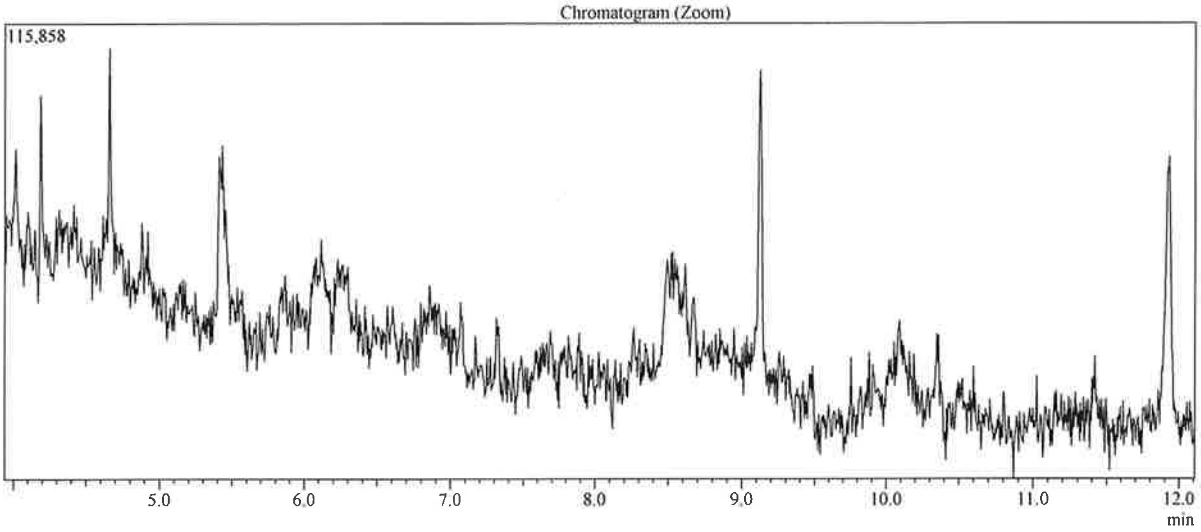

Spectrum

(4)

Line#:1 R.Time:5.430(Scan#:487)
MassPeaks:602
RawMode:Single 5.430(487) BasePeak:18.10(17414)
BG Mode:None Group 1 - Event 1

5,181

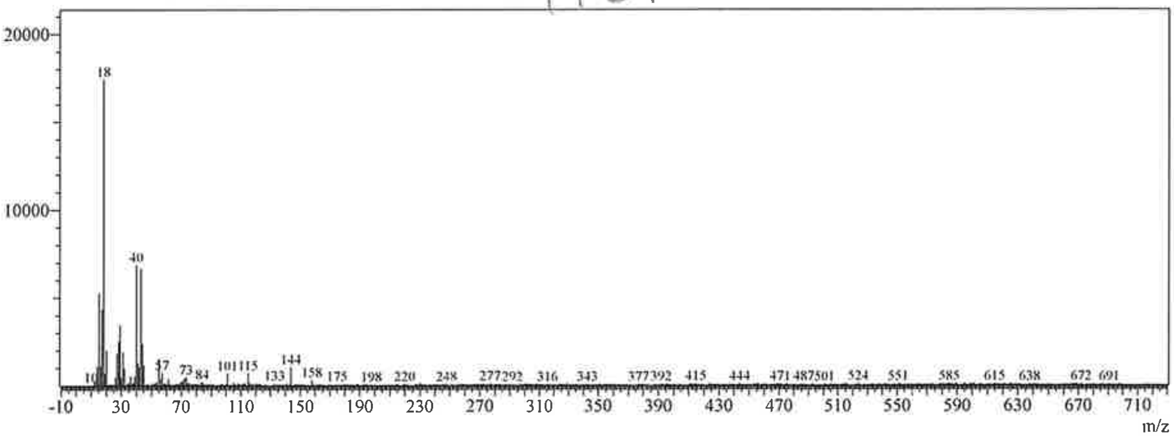

Similarity Search Results

Report View Compound Info Process Help

Hit	Similar	Regi	Compound Name	Mol Wt	Formula	Library
1	74	☑	Glycidol $$ Oxiranemethanol $$ 1-Propanol, 2,	74	C3H6O2	NIST08.LIB
2	70	☐	1-Hydroxy-2-pentanone $$ 2-Pentanone, 1-hy	102	C5H10O2	NIST08.LIB
3	69	☐	DL-Arabinose $$ Pentopyranose # $$	150	C5H10O5	NIST08.LIB
4	69	☐	Ethanamine, 2,2'-oxybis- $$ 2-(2-Aminoethoxy)	104	C4H12N2O	NIST08.LIB
5	68	☐	Glycyl-D-asparagine	189	C6H11N3O4	NIST08.LIB
6	68	☐	Oxirane, 2,3-dimethyl-, trans- $$ Butane, 2,3-e	72	C4H8O	NIST08.LIB
7	68	☐	2-Propanone, 1,3-dihydroxy- $$ Dihydroxyacet	90	C3H6O3	NIST08.LIB
8	68	☐	Oxiranemethanol, (S)- $$ (S)-(-)-Glycidol $$ 2-O	74	C3H6O2	NIST08.LIB
9	68	☐	2-Heptanol, 3-methyl- $$ 3-Methyl-2-heptanol	130	C8H18O	NIST08.LIB
10	68	☐	1,3-Butanediol $$ .beta.-Butylene glycol $$ M	90	C4H10O2	NIST08.LIB
11	68	☐	p-Dioxane, methylene- $$ 2-Methylene-1,4-dio	100	C5H8O2	NIST08.LIB

Target:

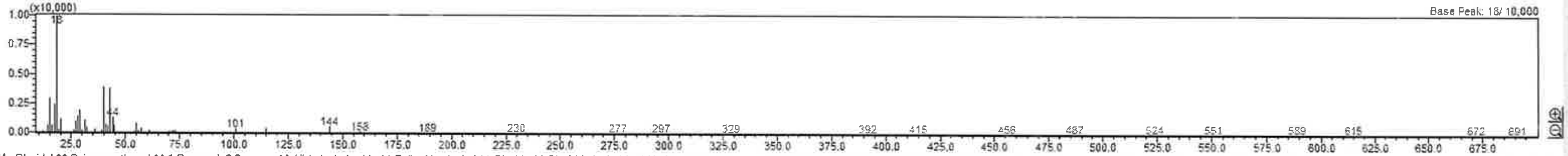

1 : 74 : Glycidol $$ Oxiranemethanol $$ 1-Propanol, 2,3-epoxy- $$ Allyl alcohol oxide $$ Epihydrin alcohol $$ Glycide $$ Glycidyl alcohol $$ 1-Hydroxy-2,3-epoxypropane $$ 1,2-Epoxy-3-hydroxypropane $$ 2-(Hydroxymethyl)oxirane $$ 2,3-Epoxy-1-propanol $$ 3-Hydroxy-1,2-epoxypropane $$ 3-Hydroxypropylene oxide $$ Hydroxymethyl

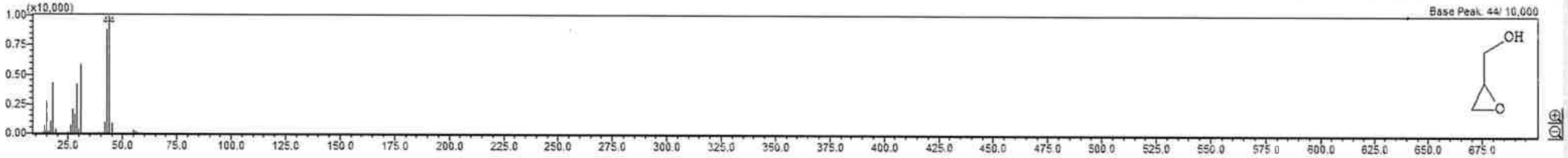

CAS#: 556-52-5 Mol Wt: 74 Serial#: 375

Cmpd Name: Glycidol $$ Oxiranemethanol $$ 1-Propanol, 2,3-epoxy- $$ Allyl alcohol oxide $$ Epihydrin alcohol $$ Glycide $$ Glycidyl alcohol $$ 1-Hydroxy-2,3-epoxypropane $$ 1,2-Epoxy-3-hydroxypropane $$ 2-(Hydroxymethyl)oxirane $$ 2,3-Epoxy-1-propanol $$ 3-Hydroxy-1,2-epoxypropane $$ 3-Hydroxypropylene oxide $$ Hydr

Formula: C3H6O2 Class Flag: No Class Flags

Ret.Index: 653

11:42 AM 2022/12/06

C:\GCMSsolution\Data\Project1\Ms Mathe\SAMPLE 3_UNKNOWN _20221130_3.qgd

Chromatogram (All TIC)]

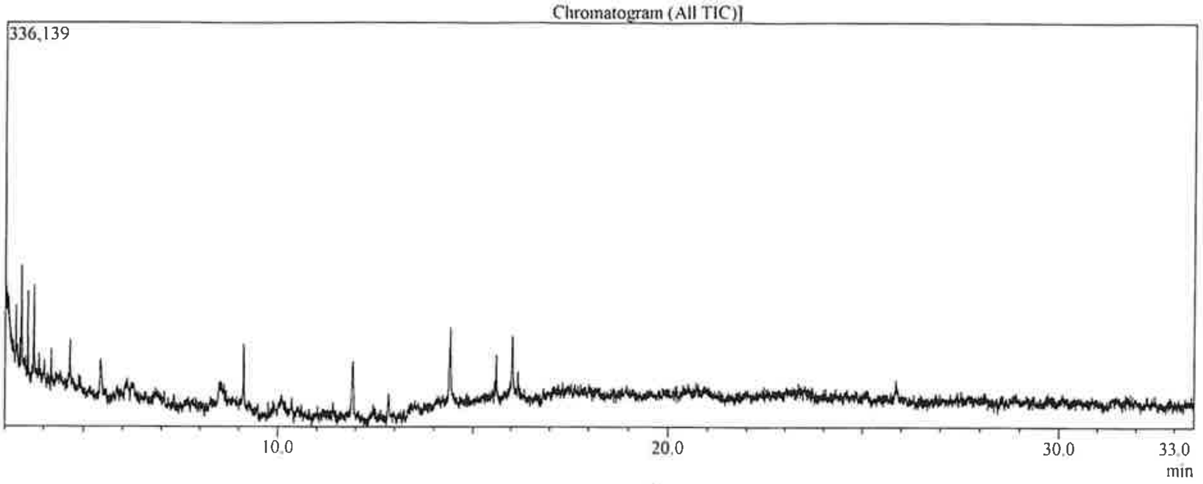

Chromatogram (Zoom)

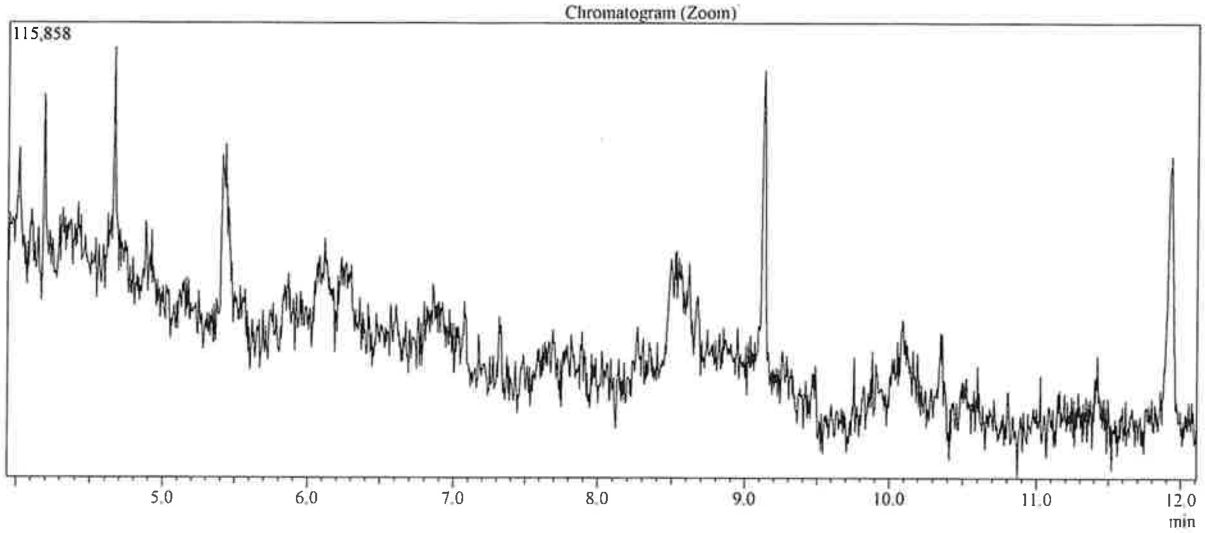

Spectrum

Line#:1 R.Time:7.070(Scan#:815)
MassPeaks:632
RawMode:Single 7.070(815) BasePeak:18.10(13660)
BG Mode:None Group 1 - Event 1

41064

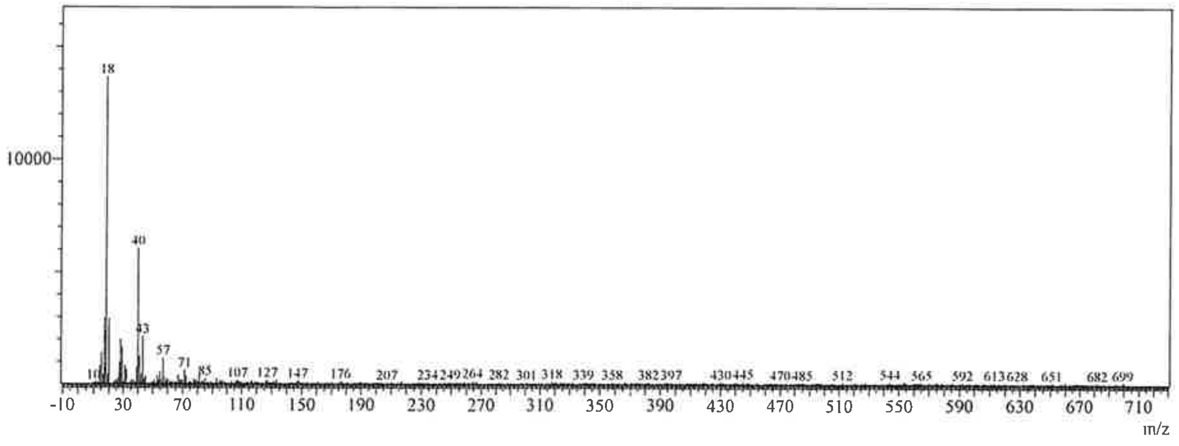

Similarity Search Results

Report View Compound Info Process Help

Hit	Similar	Regi	Compound Name	Mol Wt	Formula	Library
1	67	☑	1-Hydroxy-2-pentanone $$ 2-Pentanone, 1-hy	102	$C_5H_{10}O_2$	NIST08.LIB
2	66	☐	Glycyl-D-asparagine	189	$C_6H_{11}N_3O_4$	NIST08.LIB
3	66	☐	Glycidol $$ Oxiranemethanol $$ 1-Propanol, 2,	74	$C_3H_6O_2$	NIST08.LIB
4	65	☐	2-Butenedioic acid, 2-methyl-, (Z)- $$ Citraconi	130	$C_5H_6O_4$	NIST08.LIB
5	64	☐	2-Propanone, 1,3-dihydroxy- $$ Dihydroxyacet	90	$C_3H_6O_3$	NIST08.LIB
6	64	☐	3,3-Dimethyl-2-pentanone $$ 3,3-Dimethyl-2-p	114	$C_7H_{14}O$	NIST08.LIB
7	64	☐	anti-2-Acetoxyacetaldoxime $$ (2E)-2-(Hydroxy	117	$C_4H_7NO_3$	NIST08.LIB
8	63	☐	2-Hexanone, 5-methyl- $$ Isoamyl methyl keto	114	$C_7H_{14}O$	NIST08.LIB
9	63	☐	1-Hydroxy-3-methyl-2-butanone $$ 2-Butanone	102	$C_5H_{10}O_2$	NIST08.LIB
10	63	☐	3,4-Dimethyl-2-pentanone	114	$C_7H_{14}O$	NIST08.LIB
11	63	☐	4-Acetoxy-2-azetidinone $$ 2-Azetidinone, 4-(a	129	$C_5H_7NO_3$	NIST08.LIB

Target:

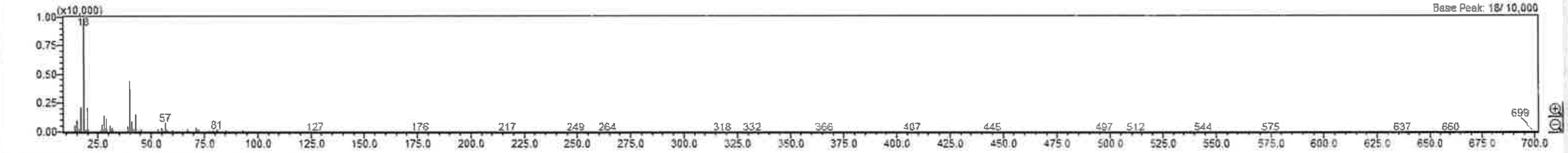

1 : 102 : 1-Hydroxy-2-pentanone $$ 2-Pentanone, 1-hydroxy- $$

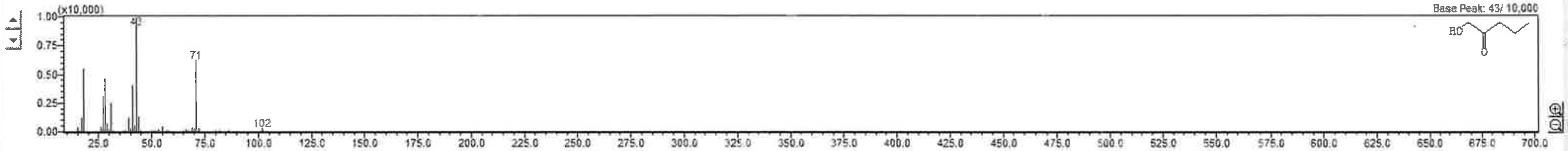

CAS#: 64502 - 89 - 2 Mol Wt: 102 Serial#: 2241

Cmpd Name: 1-Hydroxy-2-pentanone $$ 2-Pentanone, 1-hydroxy- $$

Formula: $C_5H_{10}O_2$ Class Flag: No Class Flags.

Ret.Index: 897

11:43 AM 2022/12/06

C:\GCMSsolution\Data\Project1\Ms Mathe\SAMPLE 3_UNKNOWN _20221130_3.qgd

Chromatogram (All TIC)]

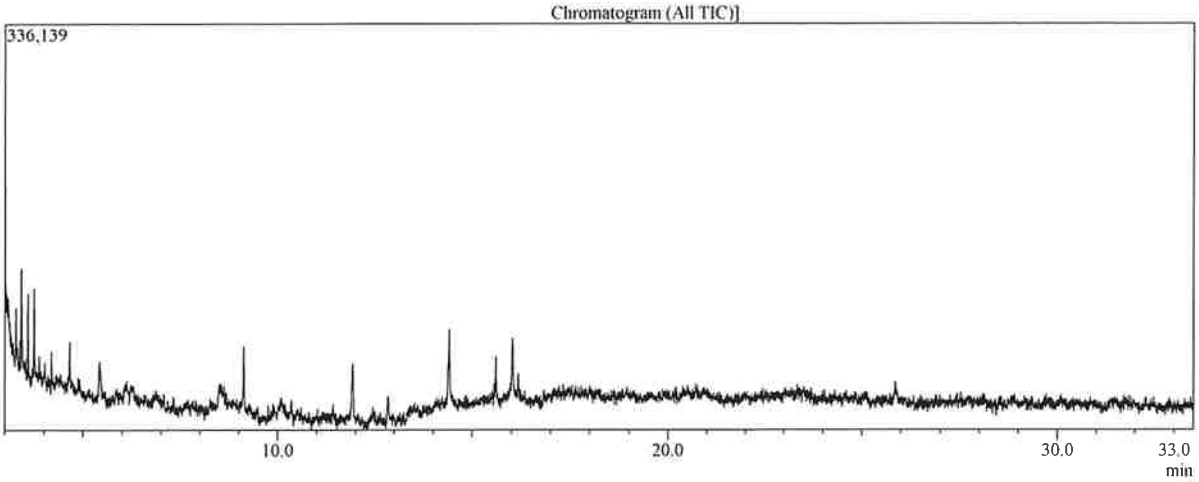

Chromatogram (Zoom)

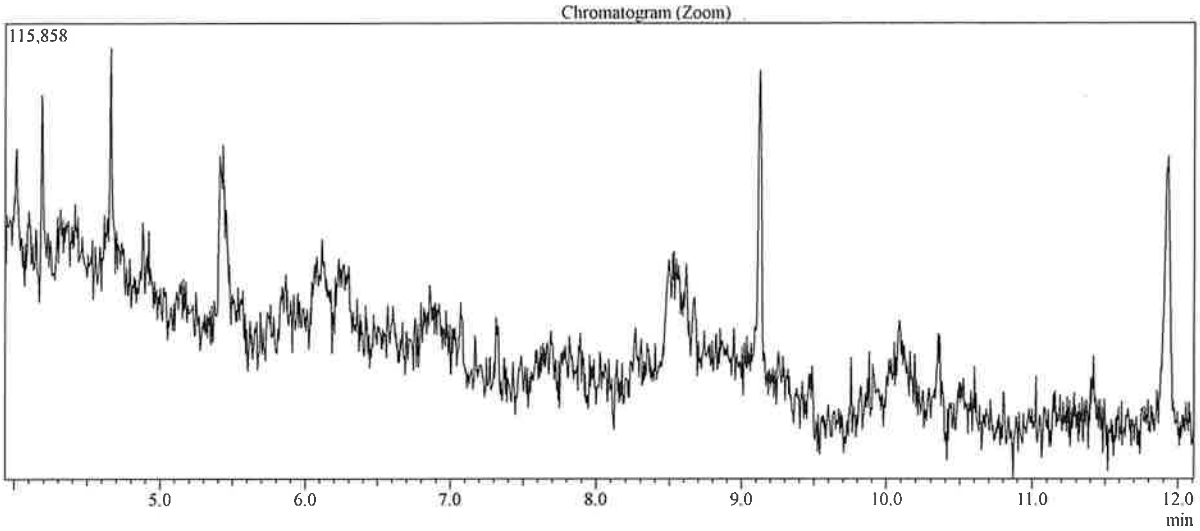

Spectrum

② 4,342

Line#:1 R.Time:7.325(Scan#:866)
MassPeaks:611
RawMode:Single 7.325(866) BasePeak:18.10(14594)
BG Mode:None Group 1 - Event 1

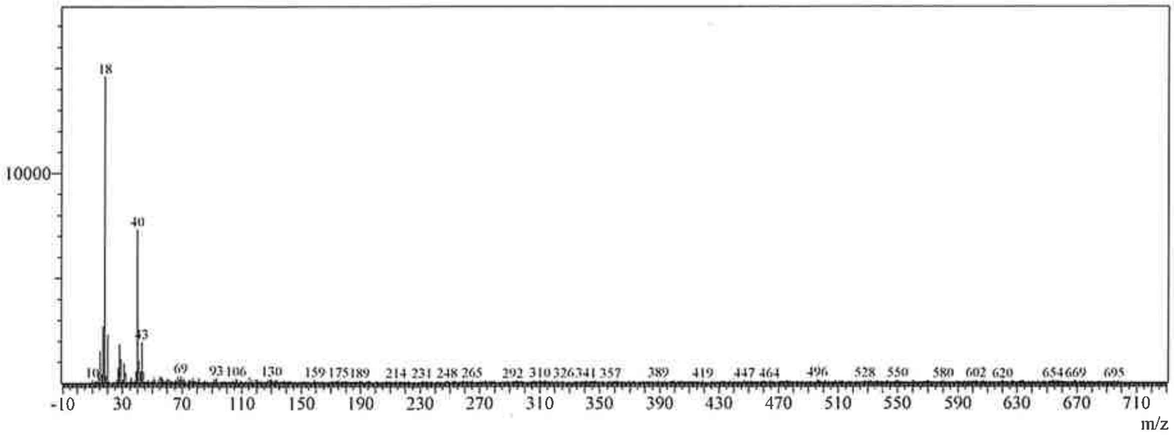

Similarity Search Results

Report View Compound Info Process Help

Hit	Similar	Regi	Compound Name	Mol Wt	Formula	Library
1	65	☑	1-Hydroxy-2-pentanone $$ 2-Pentanone, 1-hy	102	C5H10O2	NIST08.LIB
2	65	☐	Acetonitrile, hydroxy- $$ Glycolonitrile $$ Cyan	57	C2H3NO	NIST08.LIB
3	65	☐	3-Amino-1,2-propanediol $$ 1-Aminoglycerol $	91	C3H9NO2	NIST08.LIB
4	65	☐	Glycidol $$ Oxiranemethanol $$ 1-Propanol, 2,	74	C3H6O2	NIST08.LIB
5	65	☐	Glycyl-D-asparagine	189	C6H11N3O4	NIST08.LIB
6	64	☐	anti-2-Acetoxyacetaldoxime $$ (2E)-2-(Hydroxy	117	C4H7NO3	NIST08.LIB
7	63	☐	1-Propanol, 2-amino-, (+/-)- $$ 1-Propanol, 2-	75	C3H9NO	NIST08.LIB
8	63	☐	2-Butenedioic acid, 2-methyl-, (Z)- $$ Citraconi	130	C5H6O4	NIST08.LIB
9	62	☐	2-Propanone, 1,3-dihydroxy- $$ Dihydroxyacet	90	C3H6O3	NIST08.LIB
10	62	☐	3-Butyn-1-ol $$ 1-Butyn-4-ol $$ 2-Hydroxyethyl	70	C4H6O	NIST08.LIB
11	62	☐	Oxazole $$ 1,3-Oxazole $$	69	C3H3NO	NIST08.LIB

Target

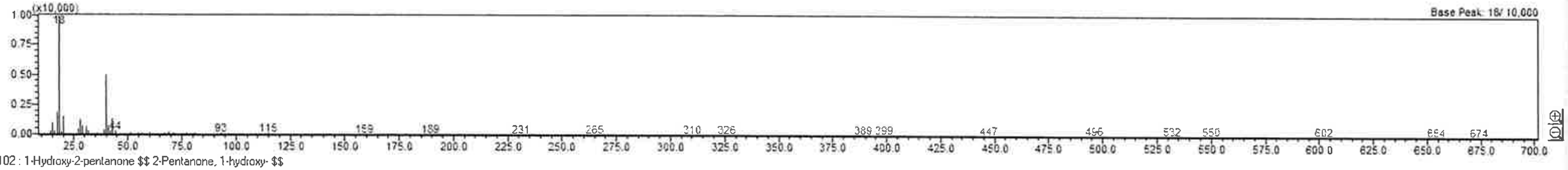

1 : 102 : 1-Hydroxy-2-pentanone $$ 2-Pentanone, 1-hydroxy- $$

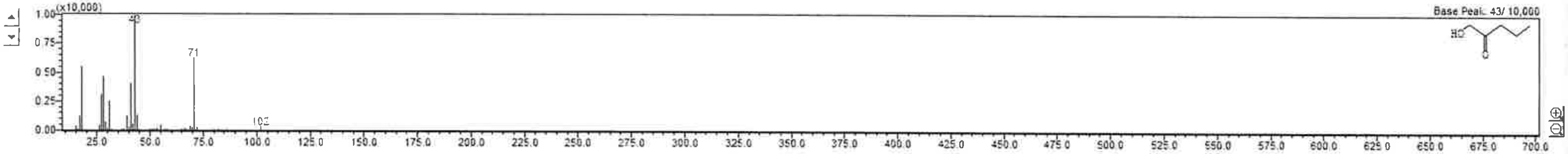

CAS#: 64502 - 89 - 2 Mol Wt: 102 Serial#: 2241

Cmpd Name: 1-Hydroxy-2-pentanone $$ 2-Pentanone, 1-hydroxy- $$

Formula: C5H10O2 Class Flag: No Class Flags

Ret.Index: 897

11:43 AM 2022/12/06

C:\GCMSsolution\Data\Project1\Ms Mathe\SAMPLE 3_UNKNOWN _20221130_3.qgd

Chromatogram (All TIC)]

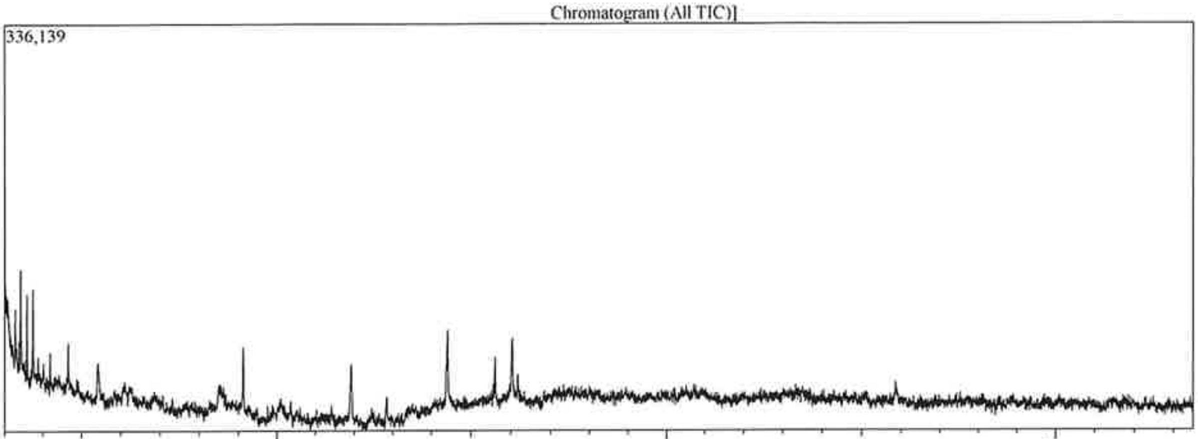

Chromatogram (Zoom)

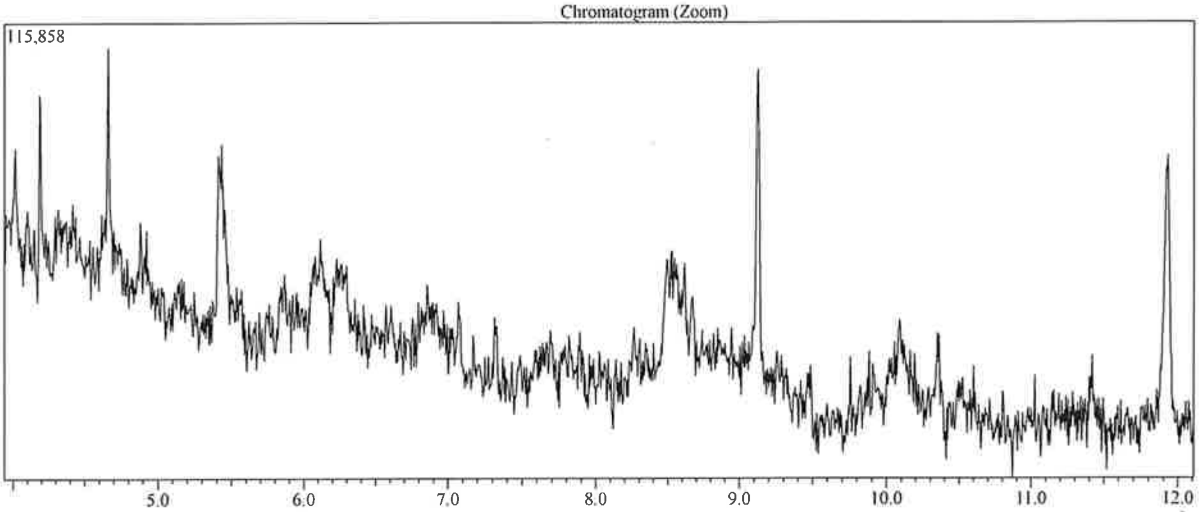

Spectrum

(4) 3,671

Line#:1 R.Time:9.135(Scan#:1228)
MassPeaks:583
RawMode:Single 9.135(1228) BasePeak:18.10(12338)
BG Mode:None Group 1 - Event 1

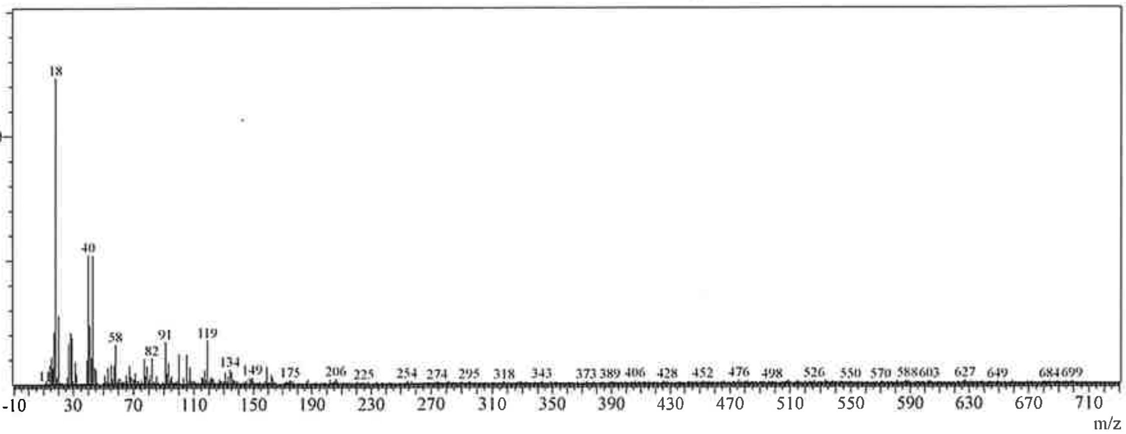

Similarity Search Results

Report View Compound Info Process Help

Hit	Similar	Regi	Compound Name	Mol Wt	Formula	Library
1	65	☑	1-Hydroxy-2-pentanone $$ 2-Pentanone, 1-hy	102	C5H10O2	NIST08.LIB
2	65	☐	Acetonitrile, hydroxy- $$ Glycolonitrile $$ Cyan	57	C2H3NO	NIST08.LIB
3	65	☐	3-Amino-1,2-propanediol $$ 1-Aminoglycerol $	91	C3H9NO2	NIST08.LIB
4	65	☐	Glycidol $$ Oxiranemethanol $$ 1-Propanol, 2,	74	C3H6O2	NIST08.LIB
5	65	☐	Glycyl-D-asparagine	189	C6H11N3O4	NIST08.LIB
6	64	☐	anti-2-Acetoxyacetaldoxime $$ (2E)-2-(Hydroxy	117	C4H7NO3	NIST08.LIB
7	63	☐	1-Propanol, 2-amino-, (+/-)- $$ 1-Propanol, 2-	75	C3H9NO	NIST08.LIB
8	63	☐	2-Butenedioic acid, 2-methyl-, (Z)- $$ Citraconi	130	C5H6O4	NIST08.LIB
9	62	☐	2-Propanone, 1,3-dihydroxy- $$ Dihydroxyacet	90	C3H6O3	NIST08.LIB
10	62	☐	3-Butyn-1-ol $$ 1-Butyn-4-ol $$ 2-Hydroxyethyl	70	C4H6O	NIST08.LIB
11	62	☐	Oxazole $$ 1,3-Oxazole $$	69	C3H3NO	NIST08.LIB

Target:

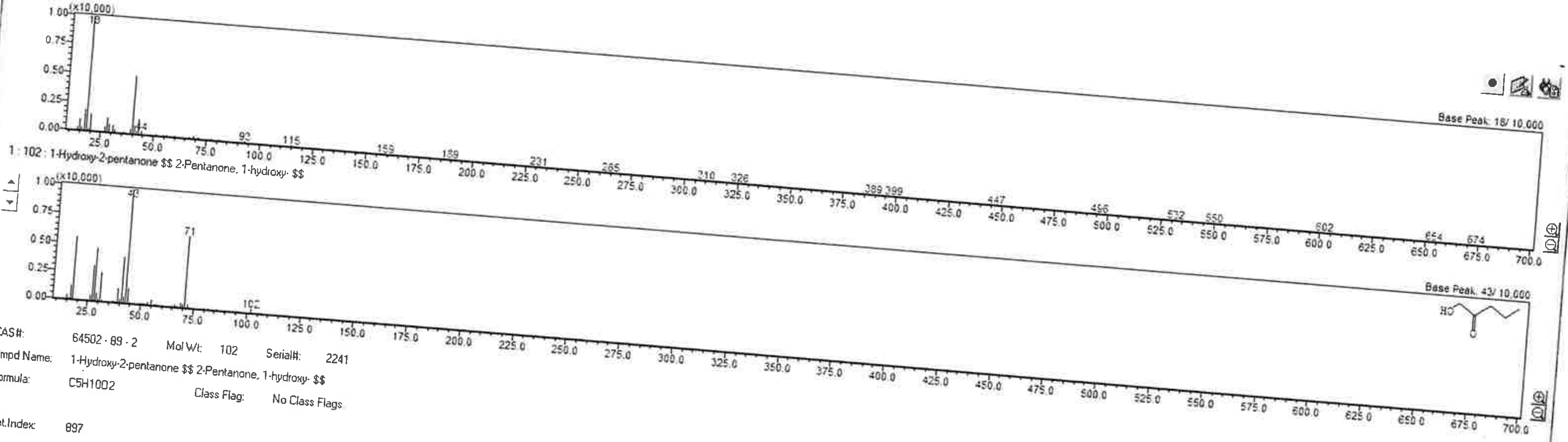

CAS#: 64502 - 89 - 2 Mol Wt: 102 Serial#: 2241

Cmpd Name: 1-Hydroxy-2-pentanone $$ 2-Pentanone, 1-hydroxy- $$

Formula: C5H10O2 Class Flag: No Class Flags

Ret.Index: 897

11:43 AM
2022/12/06

Similarity Search Results

Report View Compound Info Process Help

Hit	Similar	Regi	Compound Name	Mol Wt	Formula	Library
1	64	☑	1-Hydroxy-2-pentanone $$ 2-Pentanone, 1-hy	102	C5H10O2	NIST08.LIB
2	64	☐	Triazido-(1,2,3,4,5-pentamethylcyclopenta-2,4-	335	C10H15GeN	NIST08.LIB
3	63	☐	2-(N-Benzyloxycarbonylamino)-propanol $$ Be	209	C11H15NO3	NIST08.LIB
4	63	☐	3-Cyclohexen-1-ol, 1-methyl- $$ 1-Methyl-3-cyc	112	C7H12O	NIST08.LIB
5	62	☐	Glycidol $$ Oxiranemethanol $$ 1-Propanol, 2,	74	C3H6O2	NIST08.LIB
6	62	☐	1,3,4,6-Tetrabromopentacyclo[9.3.1.1(6,10).0(	528	C16H20Br4	NIST08.LIB
7	61	☐	7-Methyloctane-2,4-dione, enol form $$ (3Z)-4-	156	C9H16O2	NIST08.LIB
8	60	☐	4,4,6-Trimethyltetrahydro-1,3-oxazine-2-thione	159	C7H13NOS	NIST08.LIB
9	60	☐	3,3-Dimethyl-2-pentanone $$ 3,3-Dimethyl-2-p	114	C7H14O	NIST08.LIB
10	60	☐	2,4,6,8,10-Tetradecapentaenoic acid, 9a-(ace	606	C36H46O8	NIST08.LIB
11	60	☐	1,3-Dimethylcyclopentanol	114	C7H14O	NIST08.LIB

Target:

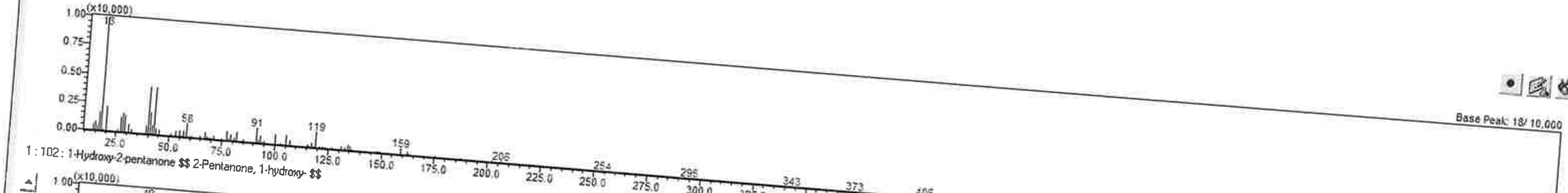

1 : 102 : 1-Hydroxy-2-pentanone $$ 2-Pentanone, 1-hydroxy- $$

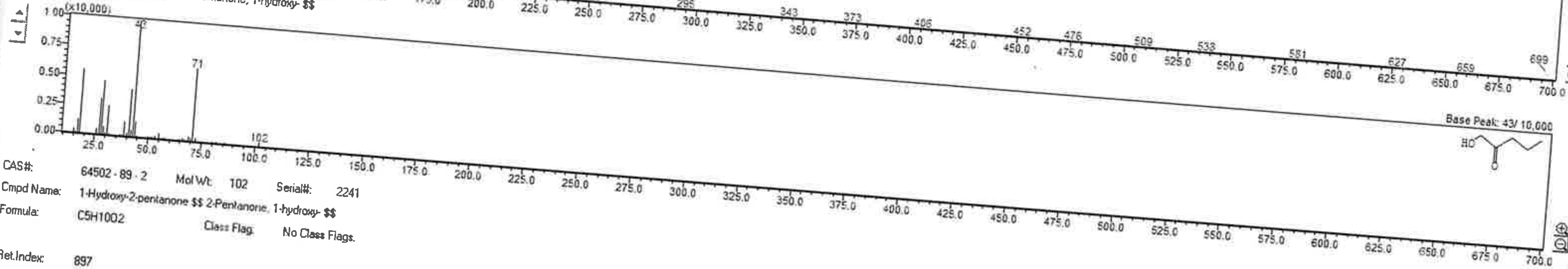

CAS#: 64502 - 89 - 2 Mol Wt: 102 Serial#: 2241

Cmpd Name: 1-Hydroxy-2-pentanone $$ 2-Pentanone, 1-hydroxy- $$

Formula: C5H10O2 Class Flag: No Class Flags.

Ret.Index: 897

11:44 AM 2022/12/06

C:\GCMSsolution\Data\Project1\Ms Mathe\SAMPLE 3_UNKNOWN _20221130_3.qgd

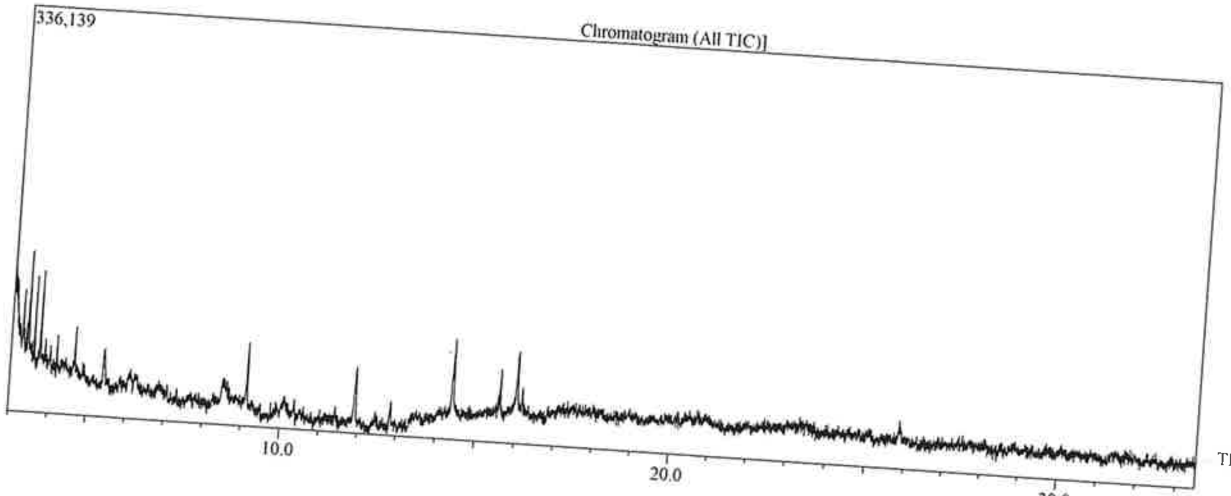

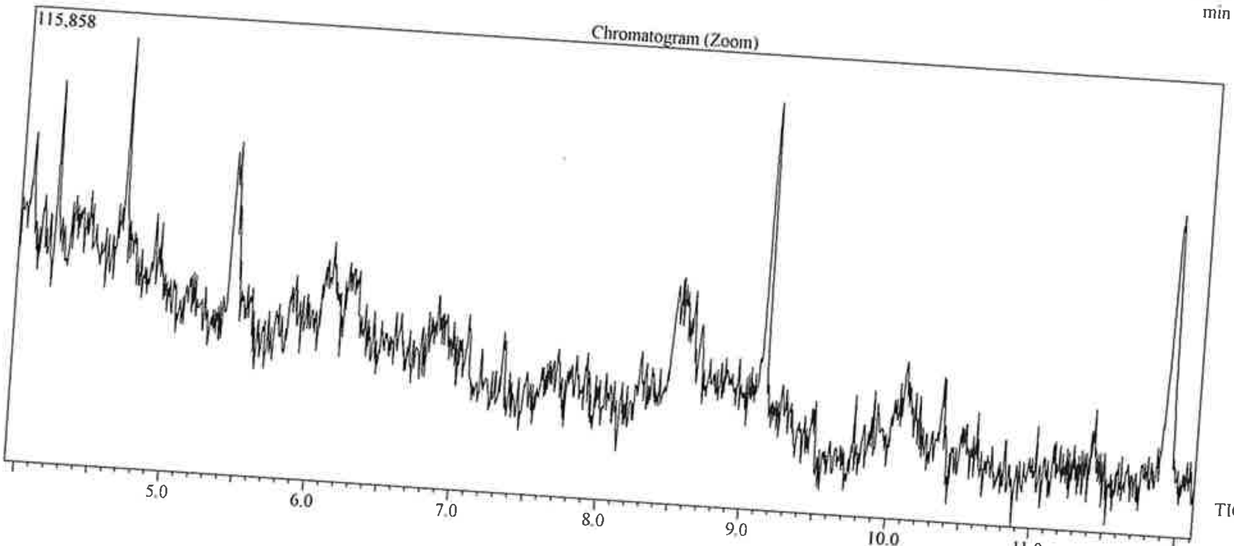

Spectrum

Line#:1 R.Time:11.925(Scan#:1786)
MassPeaks:598
RawMode:Single 11.925(1786) BasePeak:18.10(13202)
BG Mode:None Group 1 - Event 1

3,928

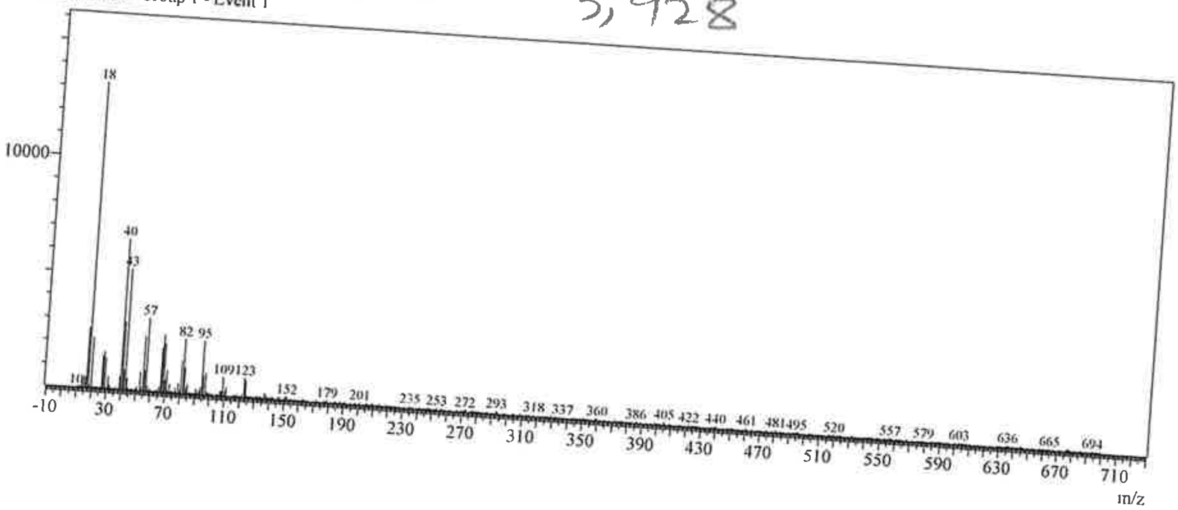

Similarity Search Results

Report View Compound Info Process Help

Hit	Similar	Regi	Compound Name	Mol Wt	Formula	Library
1	71	☑	Cyclohexanol, 3,5-dimethyl- $$ 3,5-Dimethylcy	128	C8H16O	NIST08.LIB
2	70	☐	Cyclohexanol, 3,3,5-trimethyl-, cis- $$ cis-3,3,5	142	C9H18O	NIST08.LIB
3	70	☐	3-Octen-1-ol $$ (3E)-3-Octen-1-ol # $$	128	C8H16O	NIST08.LIB
4	69	☐	11-Hexadecen-1-ol, (Z)- $$ (Z)-11-Hexadecen-	240	C16H32O	NIST08.LIB
5	69	☐	Hexanol-1, 6-cyclohexyl- $$ Cyclohexanehexa	184	C12H24O	NIST08.LIB
6	69	☐	2-Octen-1-ol $$ Oct-2-en-1-ol $$ 2-Octenol $$	128	C8H16O	NIST08.LIB
7	69	☐	2-Decen-1-ol $$ trans-2-Decen-1-ol $$ (2E)-2-	156	C10H20O	NIST08.LIB
8	69	☐	Bicyclo[2.2.2]octane-1,4-diol, monoacetate $$	184	C10H16O3	NIST08.LIB
9	69	☐	7-Octene-1,2-diol	144	C8H16O2	NIST08.LIB
10	68	☐	trans-2-Dodecen-1-ol	184	C12H24O	NIST08.LIB
11	68	☐	1-Piperidineacetonitrile $$ 1-Piperidinoacetonit	124	C7H12N2	NIST08.LIB

Target:

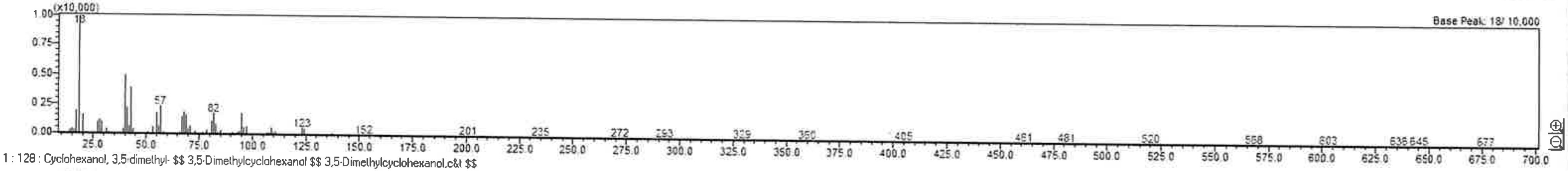

1 : 128 : Cyclohexanol, 3,5-dimethyl- $$ 3,5-Dimethylcyclohexanol $$ 3,5-Dimethylcyclohexanol,c&t $$

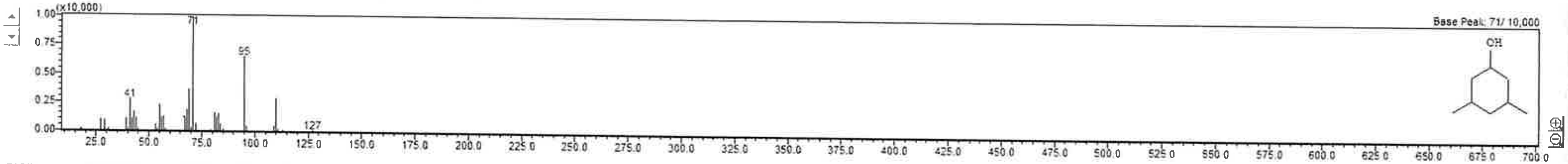

CAS#: 5441 - 52 - 1 Mol Wt: 128 Serial#: 7361

Cmpd Name: Cyclohexanol, 3,5-dimethyl- $$ 3,5-Dimethylcyclohexanol $$ 3,5-Dimethylcyclohexanol,c&t $$

Formula: C8H16O Class Flag: No Class Flags

Ret.Index: 1030

11:45 AM 2022/12/06

C:\GCMSsolution\Data\Project1\Ms Mathe\SAMPLE 3_UNKNOWN _20221130_3.qgd

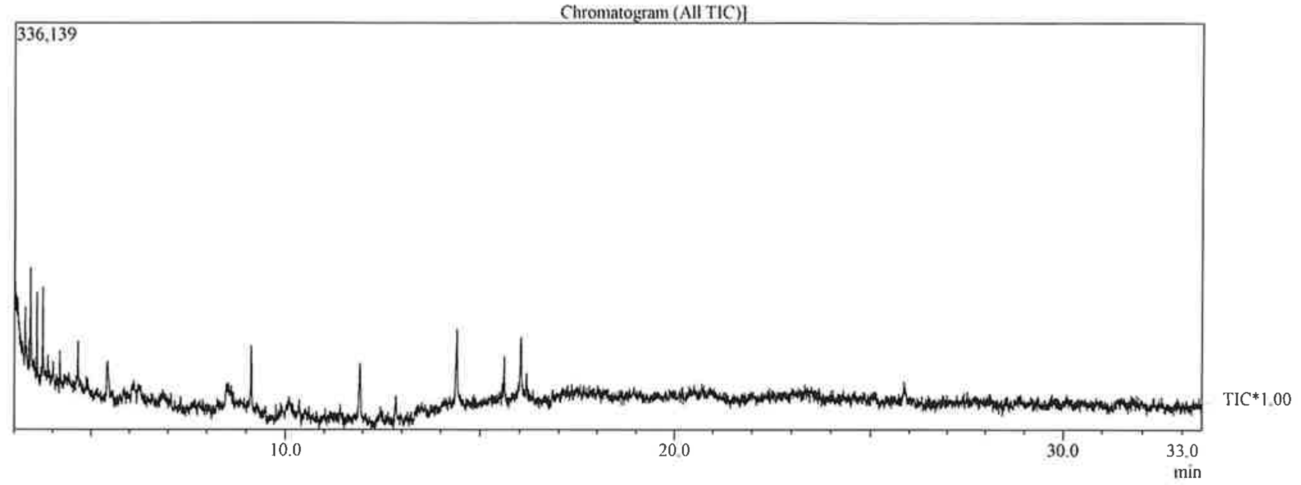

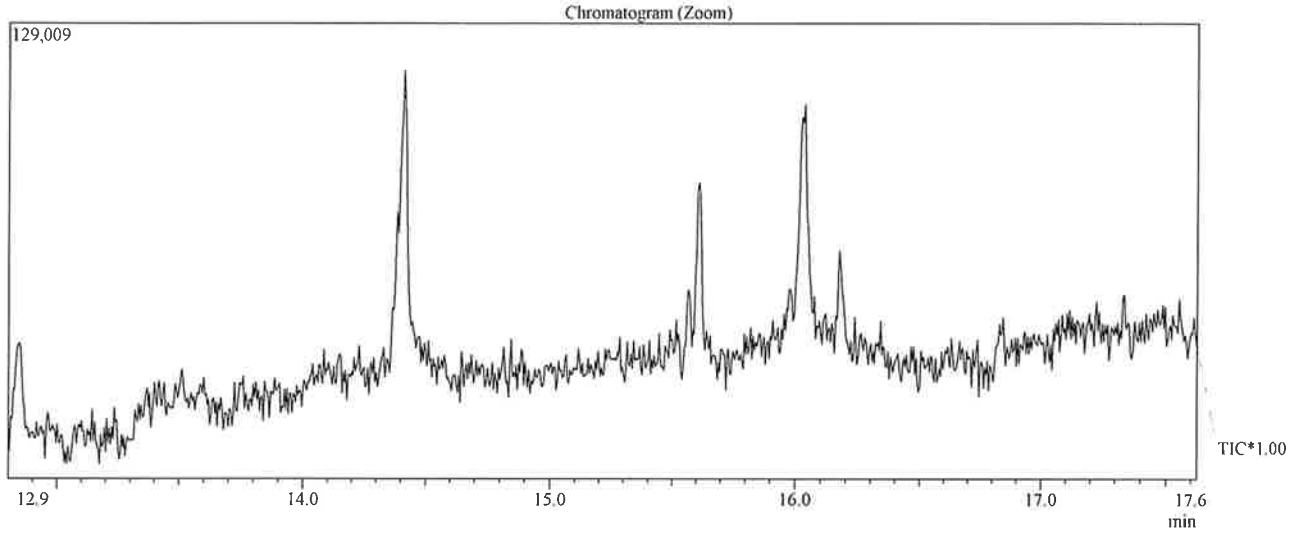

Spectrum

Line#:1 R.Time:12.850(Scan#:1971)
MassPeaks:589
RawMode:Single 12.850(1971) BasePeak:18.10(15865)
BG Mode:None Group 1 - Event 1

41720

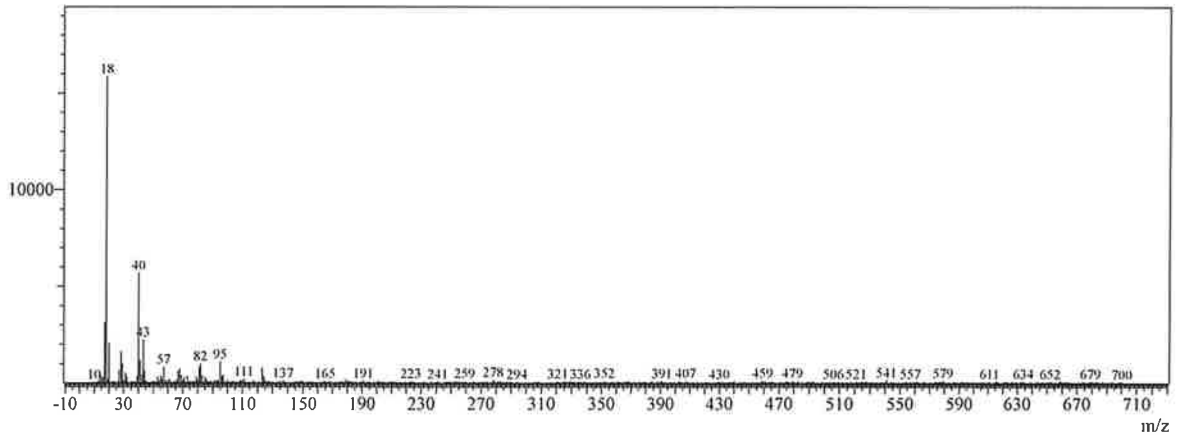

Similarity Search Results

Report View Compound Info Process Help

Hit	Similar	Regi	Compound Name	Mol Wt	Formula	Library
1	66	☑	Glycyl-D-asparagine	189	C6H11N3O4	NIST08.LIB
2	64	☐	trans-1,4-Cyclohexanediamine $$ 1,4-Diamino	114	C6H14N2	NIST08.LIB
3	63	☐	1-Hydroxy-2-pentanone $$ 2-Pentanone, 1-hy	102	C5H10O2	NIST08.LIB
4	63	☐	7-Hydroxylamino-1,3,5-triazaadamantane	170	C7H14N4O	NIST08.LIB
5	62	☐	4(4-Chlorphenyl)-3-morpholinopyrrol-2-carboxyli	320	C16H17ClN2	NIST08.LIB
6	62	☐	3-Amino-1,2-propanediol $$ 1-Aminoglycerol $	91	C3H9NO2	NIST08.LIB
7	62	☐	2-Butenedioic acid, 2-methyl-, (Z)- $$ Citraconi	130	C5H6O4	NIST08.LIB
8	62	☐	Acetonitrile, hydroxy- $$ Glycolonitrile $$ Cyan	57	C2H3NO	NIST08.LIB
9	62	☐	Cyclohexanespiro-5'-(2',4',4'-trimethyl-2'-oxazoli	181	C11H19NO	NIST08.LIB
10	61	☐	Heptanoic acid, 2-(acetyloxy)-, methyl ester $$	202	C10H18O4	NIST08.LIB
11	61	☐	DL-Arabinose $$ Pentopyranose # $$	150	C5H10O5	NIST08.LIB

Target:

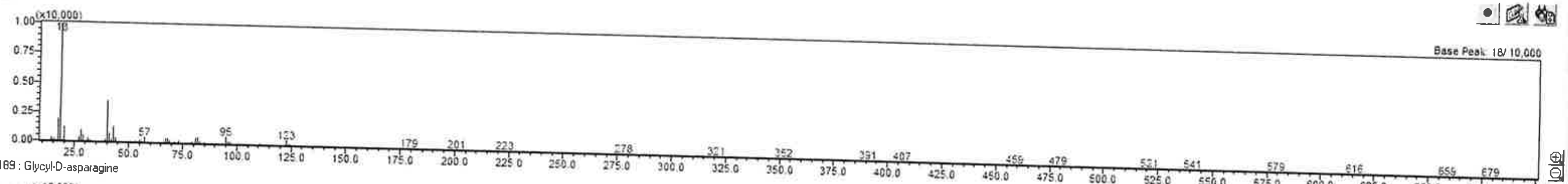

1 : 189 : Glycyl-D-asparagine

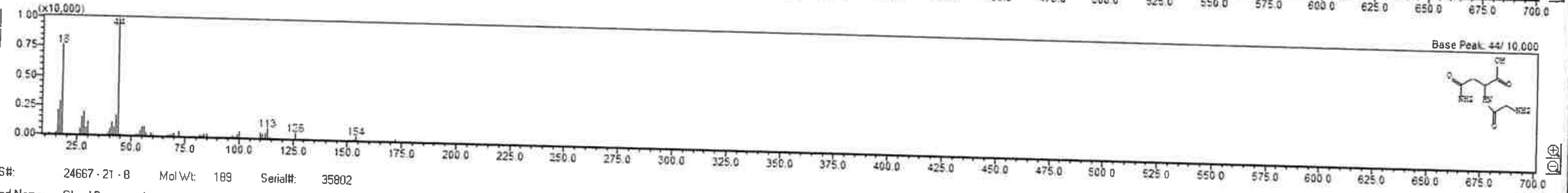

CAS#: 24667 - 21 - 8 Mol Wt: 189 Serial#: 35802

Cmpd Name: Glycyl-D-asparagine

Formula: C6H11N3O4 Class Flag: No Class Flags.

Ret.Index: 1963

11:45 AM
2022/12/06

2022/12/06 11:46:19

..\GCMSsolution\Data\Project1\Ms Mathe\SAMPLE 3_UNKNOWN _20221130_3.qgd

Chromatogram (All TIC)]

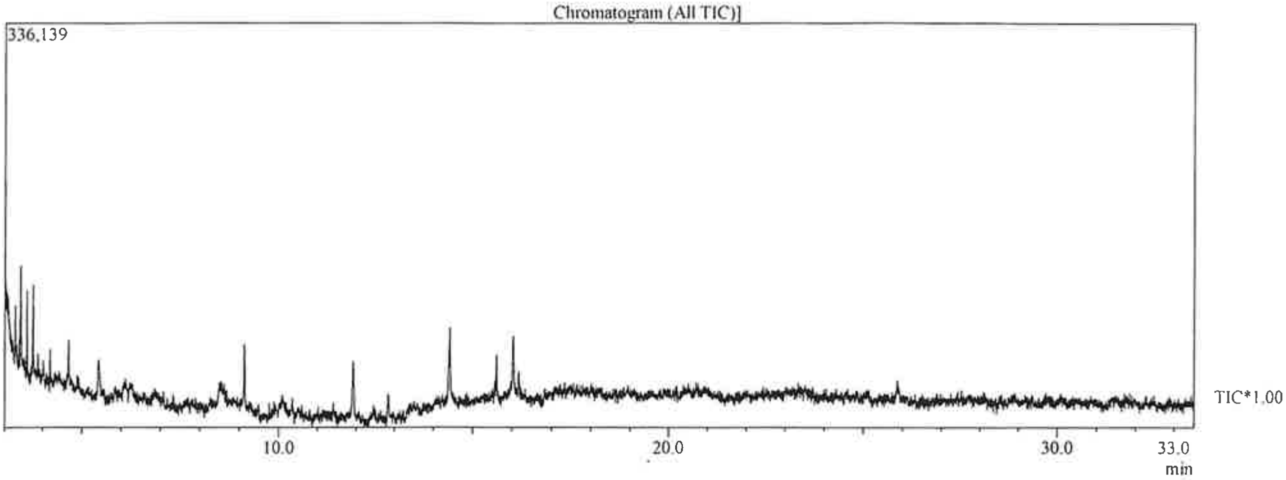

Chromatogram (Zoom)

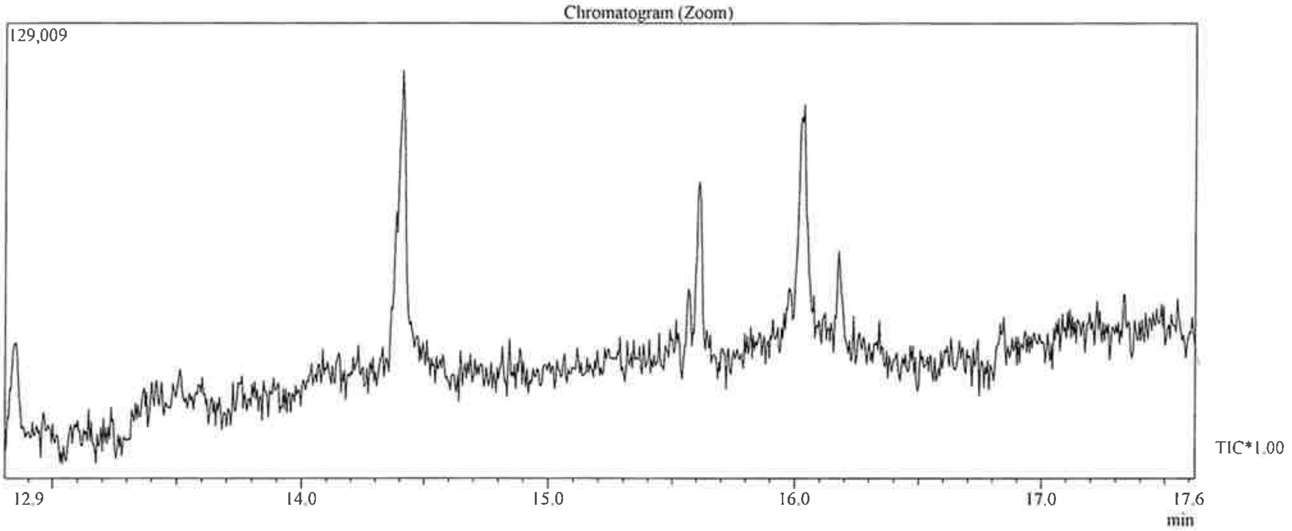

Spectrum

Line#:1 R.Time:14.410(Scan#:2283)
MassPeaks:640
RawMode:Single 14.410(2283) BasePeak:18.10(15721)
BG Mode:None Group 1 - Event 1

4677

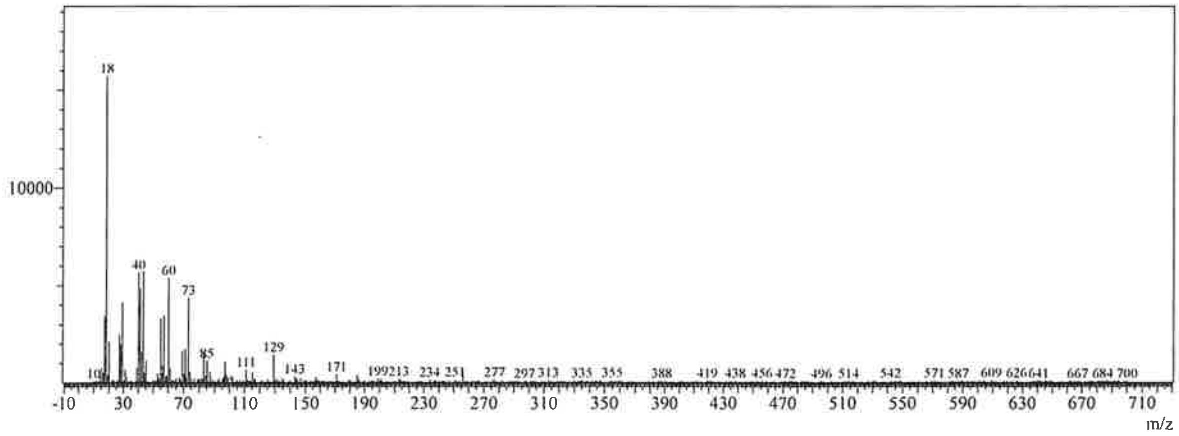

Similarity Search Results

Report View Compound Info Process Help

Hit	Similar	Regi	Compound Name	Mol Wt	Formula	Library
1	73	☑	Decanoic acid, 3-methyl- $$ 3-Methyldecanoic	186	C11H22O2	NIST08.LIB
2	72	☐	Pentanoic acid, 4-methyl- $$ Valeric acid, 4-m	116	C6H12O2	NIST08.LIB
3	72	☐	Octane, 1-propoxy- $$ Ether, octyl propyl $$ n-	172	C11H24O	NIST08.LIB
4	71	☐	3-Heptanol, 2,4-dimethyl- $$ 2,4-Dimethyl-3-he	144	C9H20O	NIST08.LIB
5	71	☐	Nonanedioic acid, dihexyl ester $$ Azelaic aci	356	C21H40O4	NIST08.LIB
6	71	☐	3-Octanol, 3,6-dimethyl- $$ AR 1 $$ 3,6-Dimet	158	C10H22O	NIST08.LIB
7	71	☐	DL-Arabinose $$ Pentopyranose # $$	150	C5H10O5	NIST08.LIB
8	71	☐	.beta.-D-Glucopyranose $$ Hexopyranose # $	180	C6H12O6	NIST08.LIB
9	70	☐	L-Galactose, 6-deoxy- $$ 6-Deoxyhexose # $$	164	C6H12O5	NIST08.LIB
10	70	☐	3-Hexanol, 3-methyl- $$ 2-Ethyl-2-pentanol $$	116	C7H16O	NIST08.LIB
11	70	☐	D-Glucopyranuronic acid $$ Glucopyranuronic	194	C6H10O7	NIST08.LIB

Target:

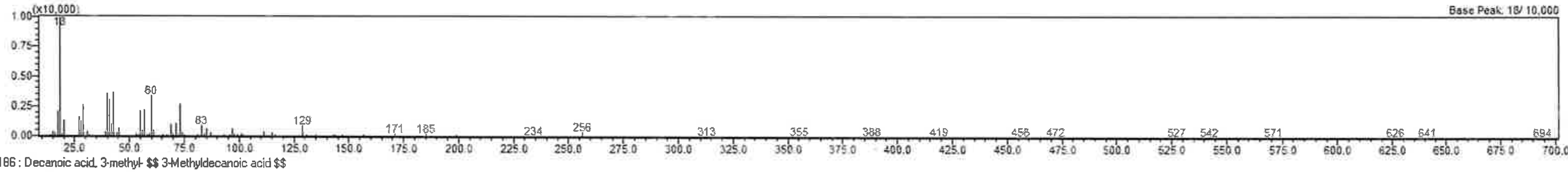

1 : 186 : Decanoic acid, 3-methyl- $$ 3-Methyldecanoic acid $$

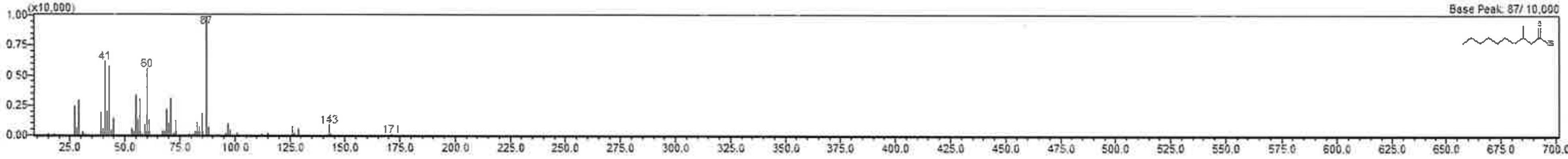

CAS#: 60308 - 82 - 9 Mol Wt: 186 Serial#: 34564

Cmpd Name: Decanoic acid, 3-methyl- $$ 3-Methyldecanoic acid $$

Formula: C11H22O2 Class Flag: No Class Flags.

Ret.Index: 1407

11:46 AM 2022/12/06

C:\GCMSsolution\Data\Project1\Ms Mathe\SAMPLE 3_UNKNOWN _20221130_3.qgd

Chromatogram (All TIC)]

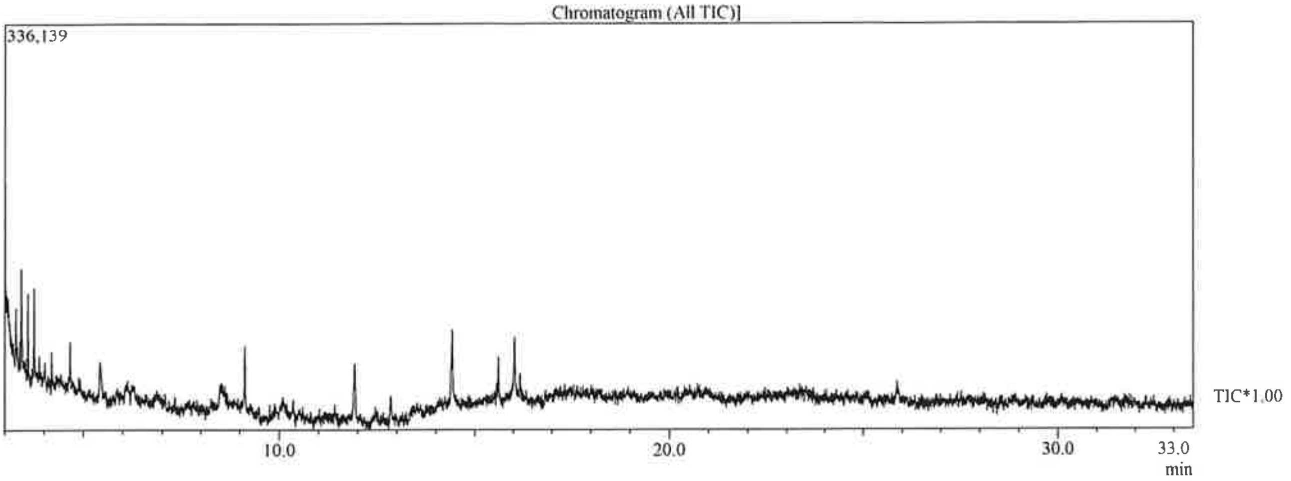

Chromatogram (Zoom)

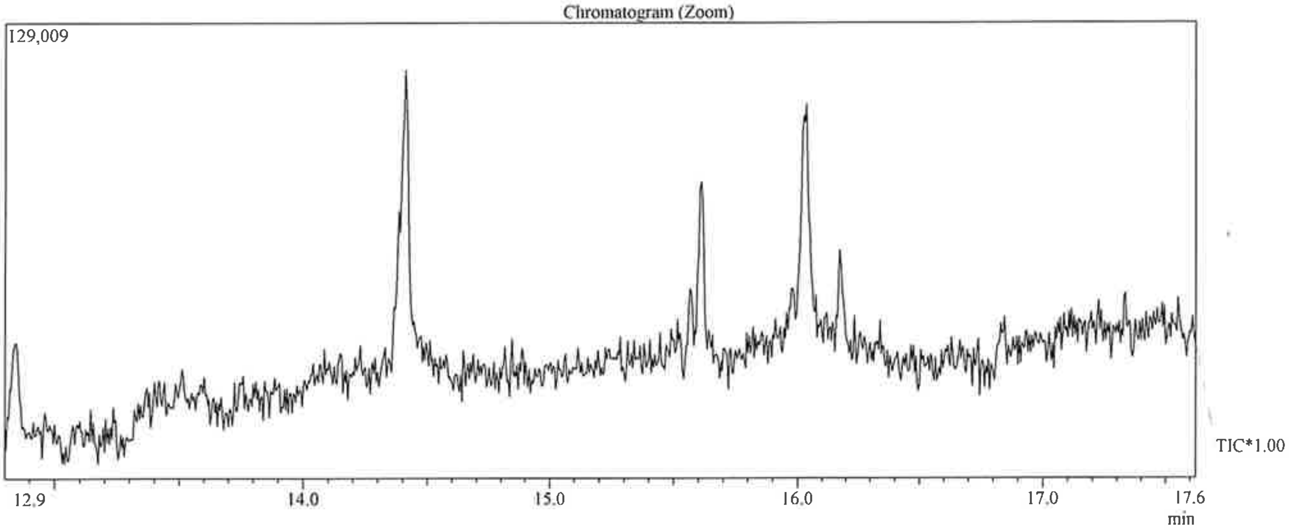

Spectrum

Line#:1 R.Time:15.575(Scan#:2516)
MassPeaks:631
RawMode:Single 15.575(2516) BasePeak:18.10(19174)
BG Mode:None Group 1 - Event 1

5,704

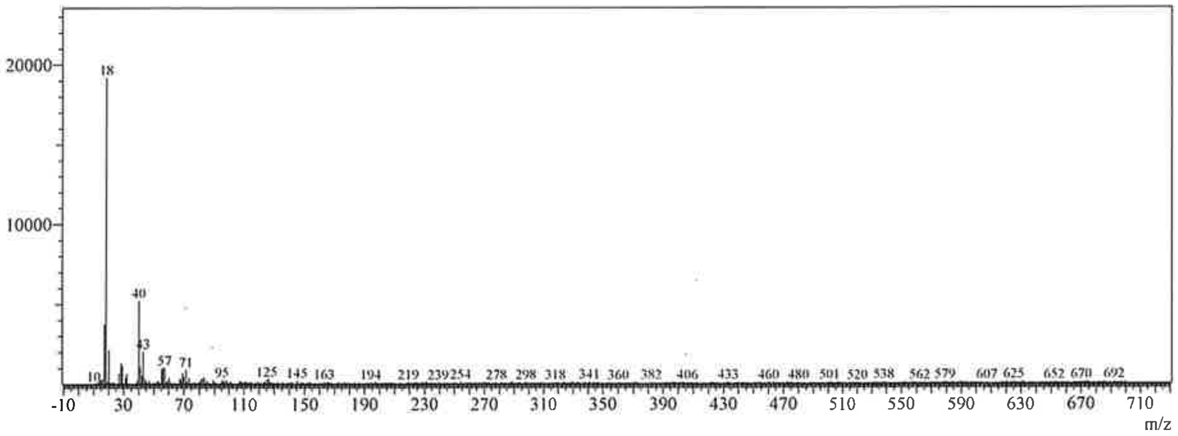

Similarity Search Results

Report View Compound Info Process Help

Hit	Similar	Regi	Compound Name	Mol Wt	Formula	Library
1	68	☑	4(4-Chlorphenyl)-3-morpholinopyrrol-2-carboxyli	320	C16H17ClN2	NIST08.LIB
2	67	☐	Glycyl-D-asparagine	189	C6H11N3O4	NIST08.LIB
3	64	☐	Water $$ Water vapor $$ Distilled water $$ Ic	18	H2O	NIST08.LIB
4	64	☐	1-Hydroxy-2-pentanone $$ 2-Pentanone, 1-hy	102	C5H10O2	NIST08.LIB
5	63	☐	Heptanoic acid, 2-(acetyloxy)-, methyl ester $$	202	C10H18O4	NIST08.LIB
6	62	☐	Guanidine $$ Aminoformamidine $$ Aminomet	59	CH5N3	NIST08.LIB
7	61	☐	Glycidol $$ Oxiranemethanol $$ 1-Propanol, 2,	74	C3H6O2	NIST08.LIB
8	61	☐	2-Azetidinone, 3,3-dimethyl- $$ Pivalolactam $	99	C5H9NO	NIST08.LIB
9	61	☐	3,4,4,6-Tetramethyltetrahydro-1,3-oxazin-2-one	157	C8H15NO2	NIST08.LIB
10	61	☐	2-Butenedioic acid, 2-methyl-, (Z)- $$ Citraconi	130	C5H6O4	NIST08.LIB
11	60	☐	anti-2-Acetoxyacetaldoxime $$ (2E)-2-(Hydroxy	117	C4H7NO3	NIST08.LIB

Target:

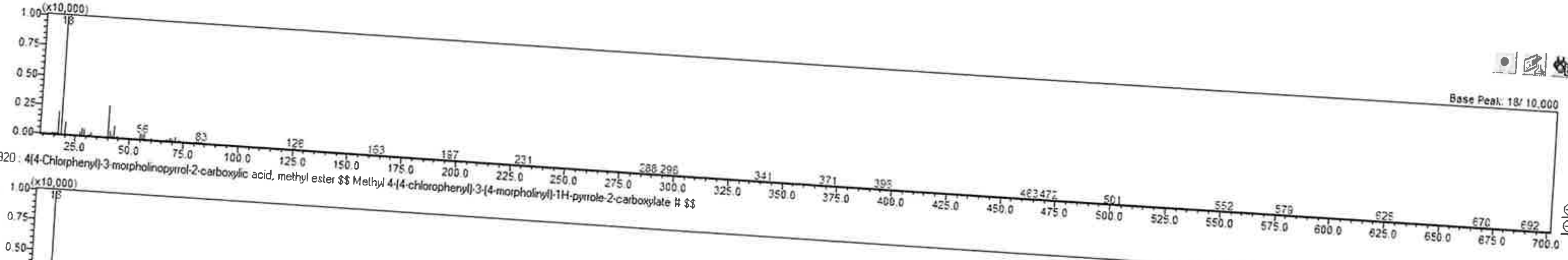

1 : 320 : 4(4-Chlorphenyl)-3-morpholinopyrrol-2-carboxylic acid, methyl ester $$ Methyl 4-(4-chlorophenyl)-3-(4-morpholinyl)-1H-pyrrole-2-carboxylate # $$

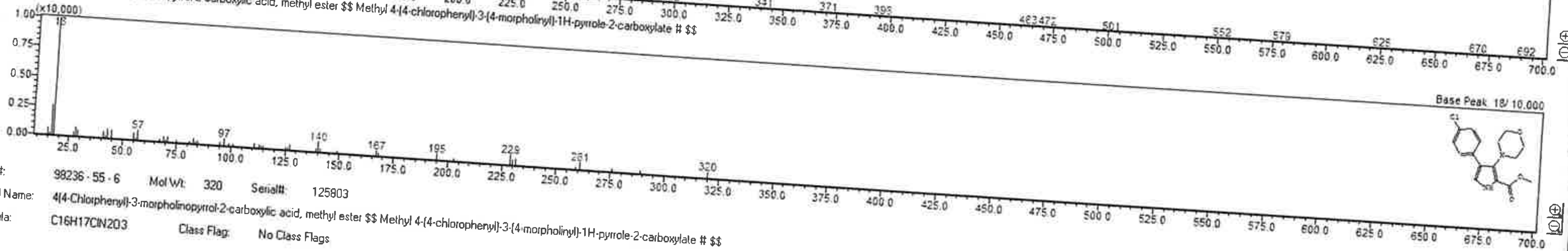

CAS#: 98236 - 55 - 6 Mol Wt: 320 Serial#: 125803

Cmpd Name: 4(4-Chlorphenyl)-3-morpholinopyrrol-2-carboxylic acid, methyl ester $$ Methyl 4-(4-chlorophenyl)-3-(4-morpholinyl)-1H-pyrrole-2-carboxylate # $$

Formula: C16H17ClN2O3 Class Flag: No Class Flags

Ret Index: 2600

11:46 AM
2022/12/06

C:\GCMSsolution\Data\Project1\Ms Mathe\SAMPLE 3_UNKNOWN _20221130_3.qgd

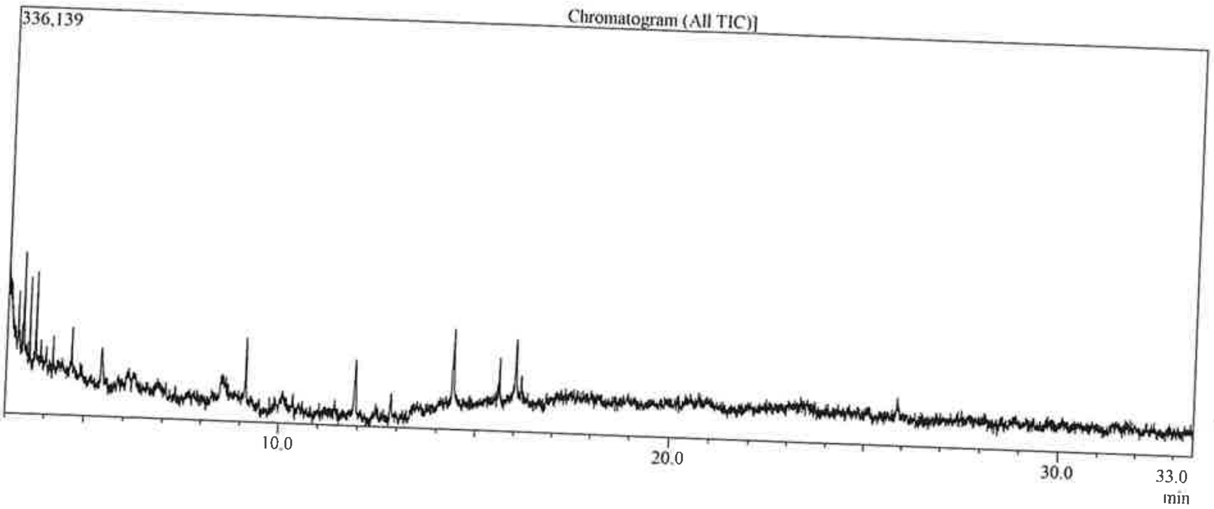

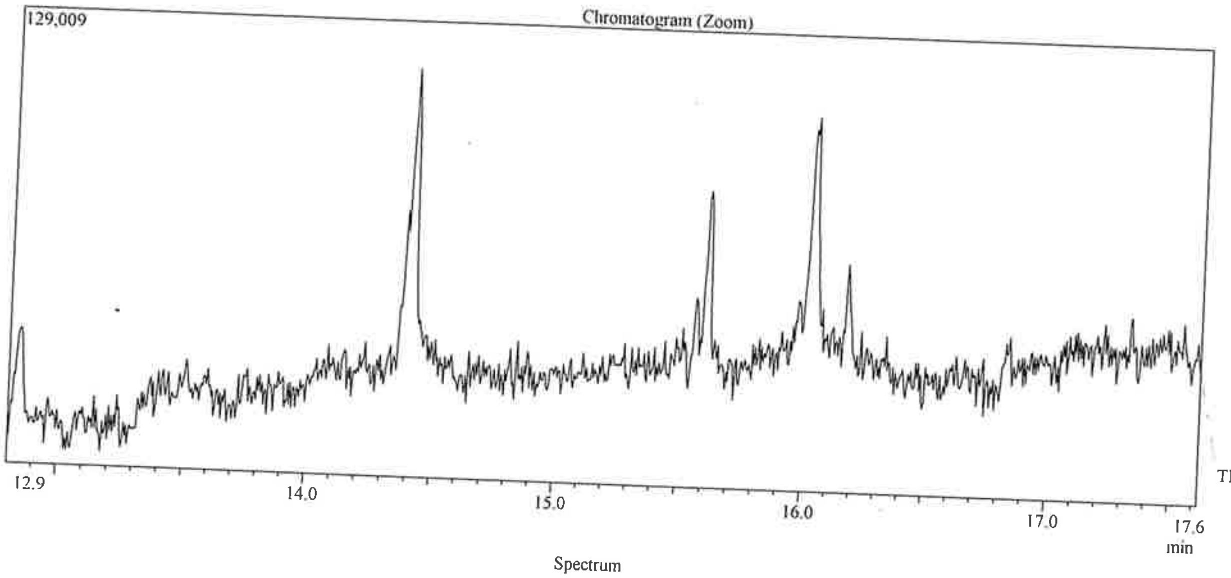

Spectrum

Line#:1 R.Time:15.610(Scan#:2523)
MassPeaks:627
RawMode:Single 15.610(2523) BasePeak:18.10(18518)
BG Mode:None Group 1 - Event 1

51509

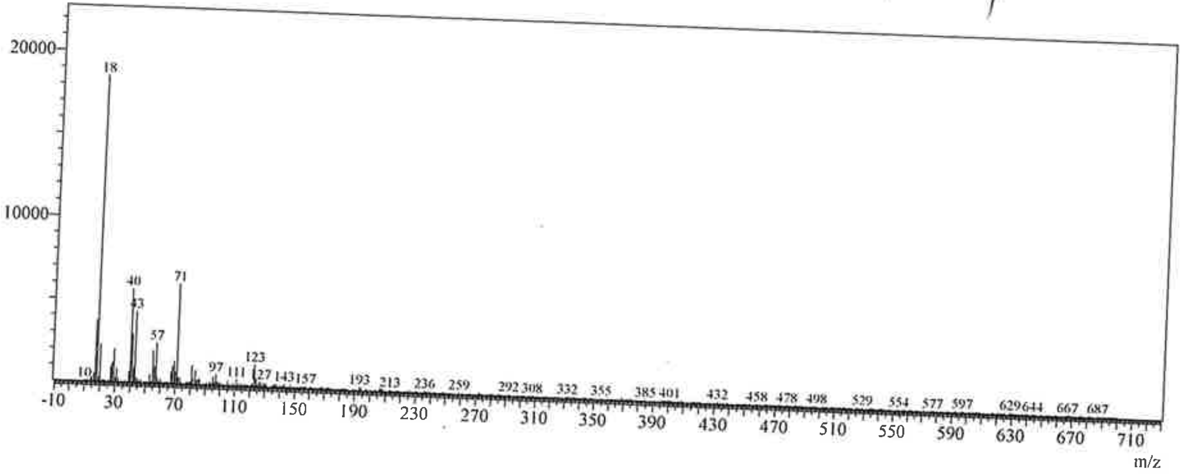

Similarity Search Results

Report View Compound Info Process Help

Hit	Similar	Regi	Compound Name	Mol Wt	Formula	Library
1	69	☑	1-Hydroxy-2-pentanone $$ 2-Pentanone, 1-hy	102	C5H10O2	NIST08.LIB
2	68	☐	Pantolactone $$ 2(3H)-Furanone, dihydro-3-hy	130	C6H10O3	NIST08.LIB
3	68	☐	Furan, 2-butyltetrahydro- $$ Octane, 1,4-epoxy	128	C8H16O	NIST08.LIB
4	68	☐	Pentanoic acid, 2,2,4-trimethyl-3-hydroxy-, isob	216	C12H24O3	NIST08.LIB
5	67	☐	l-Pantoyl lactone $$ 3-Hydroxy-4,4-dimethyldih	130	C6H10O3	NIST08.LIB
6	67	☐	3,3-Dimethyl-2-pentanone $$ 3,3-Dimethyl-2-p	114	C7H14O	NIST08.LIB
7	67	☐	Glycyl-D-asparagine	189	C6H11N3O4	NIST08.LIB
8	66	☐	2-Hepten-4-ol $$ (2E)-2-Hepten-4-ol # $$	114	C7H14O	NIST08.LIB
9	66	☐	1-Pentanol, 2-methyl- $$ 2-Methyl-1-pentanol $	102	C6H14O	NIST08.LIB
10	66	☐	Octane, 1-propoxy- $$ Ether, octyl propyl $$ n-	172	C11H24O	NIST08.LIB
11	66	☐	4-(4-Chlorophenyl)-3-morpholinopyrrol-2-carboxyli	320	C16H17ClN2	NIST08.LIB

Target

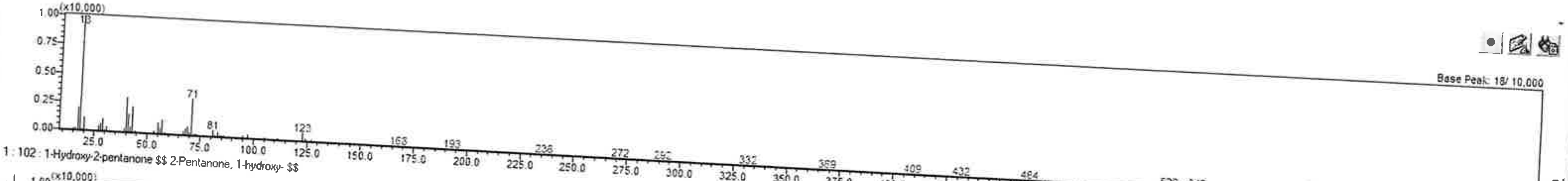

1 : 102 : 1-Hydroxy-2-pentanone $$ 2-Pentanone, 1-hydroxy- $$

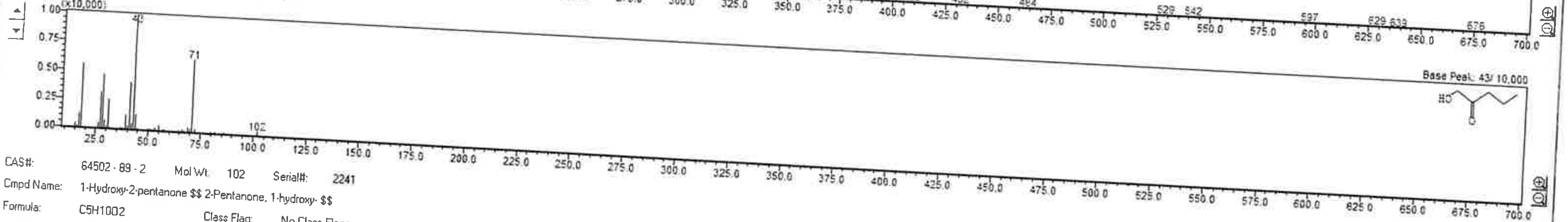

CAS#: 64502 - 89 - 2 Mol Wt: 102 Serial#: 2241

Cmpd Name: 1-Hydroxy-2-pentanone $$ 2-Pentanone, 1-hydroxy- $$

Formula: C5H10O2 Class Flag: No Class Flags

Ret.Index: 897

11:48 AM
2022/12/06

C:\GCMSsolution\Data\Project1\Ms Mathe\SAMPLE 3_UNKNOWN _20221130_3.qgd

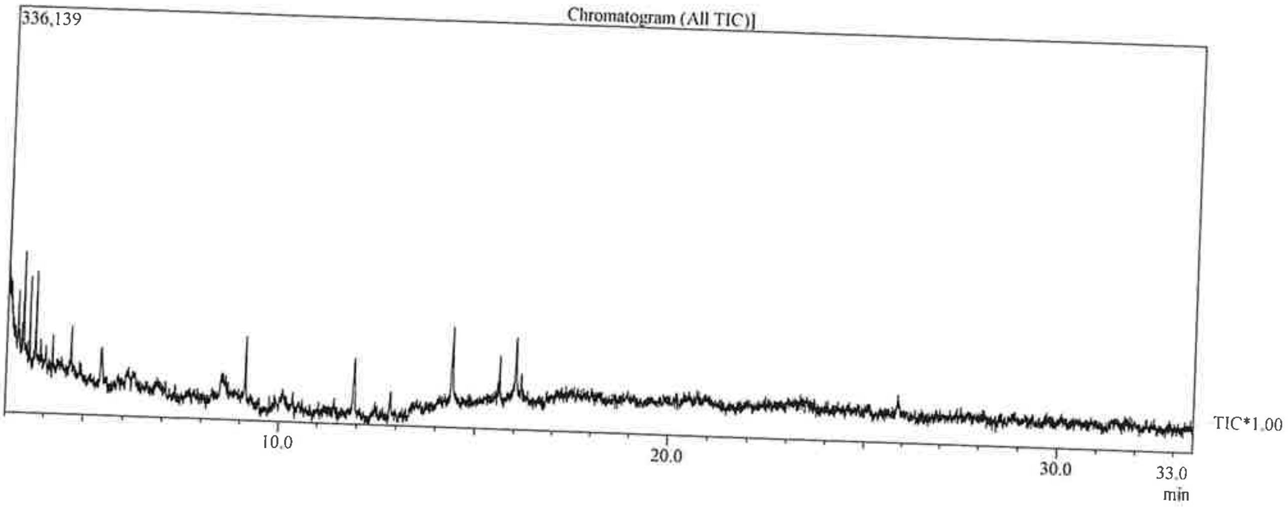

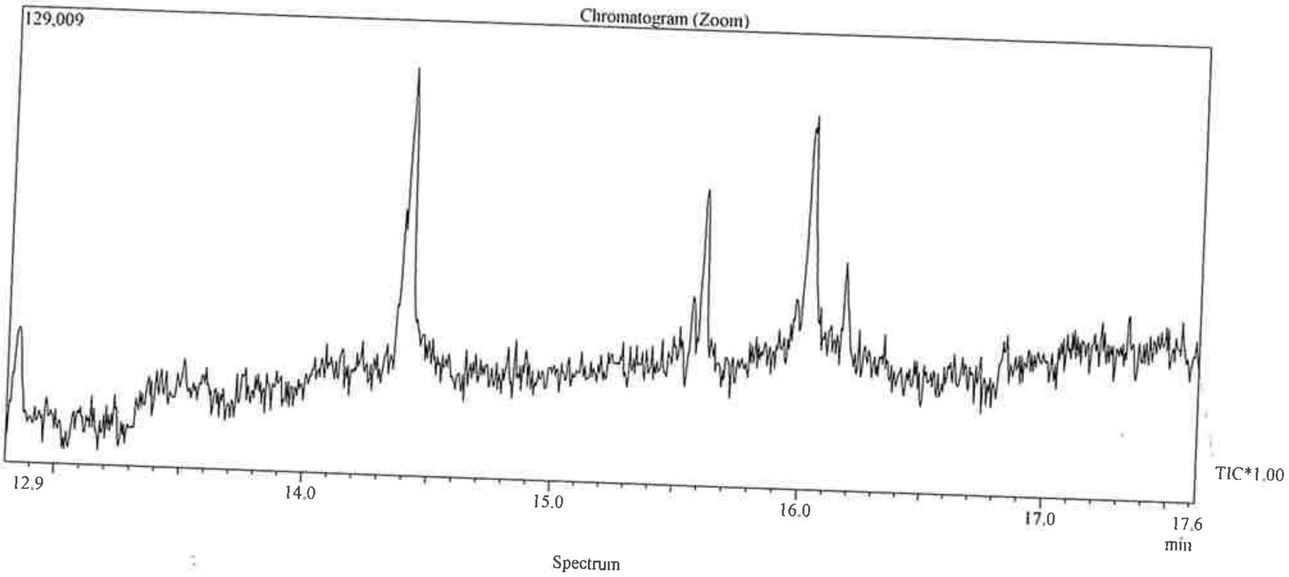

Spectrum

Line#:1 R.Time:16.040(Scan#:2609)
MassPeaks:624
RawMode:Single 16.040(2609) BasePeak:18.15(18369)
BG Mode:None Group 1 - Event 1

S1465

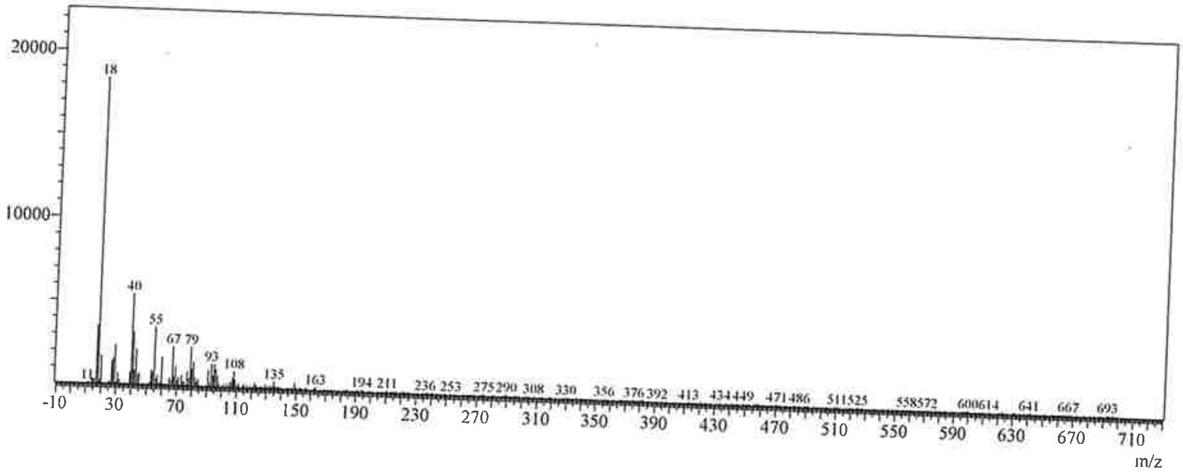

Similarity Search Results

Report View Compound Info Process Help

Hit	Similar	Regi	Compound Name	Mol Wt	Formula	Library
1	69	☑	1,4-Cyclohexanedimethanol $$ 1,4-Bis(hydroxy	144	C8H16O2	NIST08.LIB
2	66	☐	7-Octene-1,2-diol	144	C8H16O2	NIST08.LIB
3	65	☐	2,4,6,8,10-Tetradecapentaenoic acid, 9a-(ace	606	C36H46O8	NIST08.LIB
4	64	☐	2,7-Octadien-1-ol $$ 1-Octa-2,7-dienol $$ (2E)	126	C8H14O	NIST08.LIB
5	64	☐	1-((5-Oxopyrrolidin-2-yl)amino)-adamantane	234	C14H22N2O	NIST08.LIB
6	64	☐	Bicyclo[2.2.1]heptan-2-ol, endo- $$ 2-Norborn	112	C7H12O	NIST08.LIB
7	64	☐	2,4-Heptadien-1-ol, (E,E)- $$ (E,E)-2,4-Heptadi	112	C7H12O	NIST08.LIB
8	64	☐	Cycloheptanemethanol $$ Cycloheptylmethan	128	C8H16O	NIST08.LIB
9	64	☐	3-Heptyn-1-ol	112	C7H12O	NIST08.LIB
10	64	☐	cis-Hexenyl octyne carbonate $$ 2-Nonynoic	236	C15H24O2	NIST08.LIB
11	64	☐	2,4-Nonadien-1-ol $$ Nonadien-1-ol $$ (2E,4E	140	C9H16O	NIST08.LIB

Target:

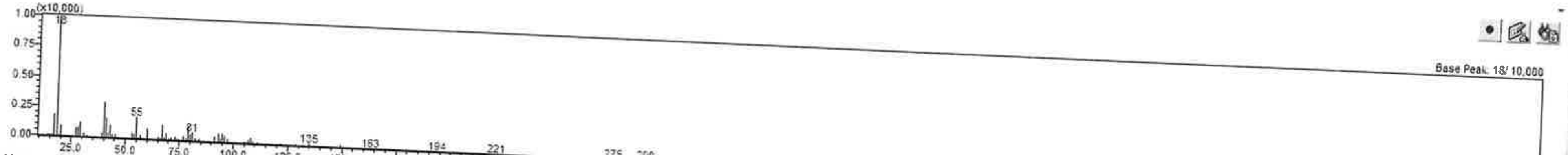

1 : 144 : 1,4-Cyclohexanedimethanol $$ 1,4-Bis(hydroxymethyl)cyclohexane $$ 1,4-Dimethylolcyclohexane $$ 1,4-Cyclohexanedimethanol,c&t $$ 1,4-Bis(hydroxymethyl)cyclohexane,c&t $$ Cyclohexane-1,4-dimethanol,c&t $$ trans-1,4-Cyclohexanedimethanol $$ CHDM $$ 1,4-Chidm $$ Cyclohexane-1,4-dimethanol $$

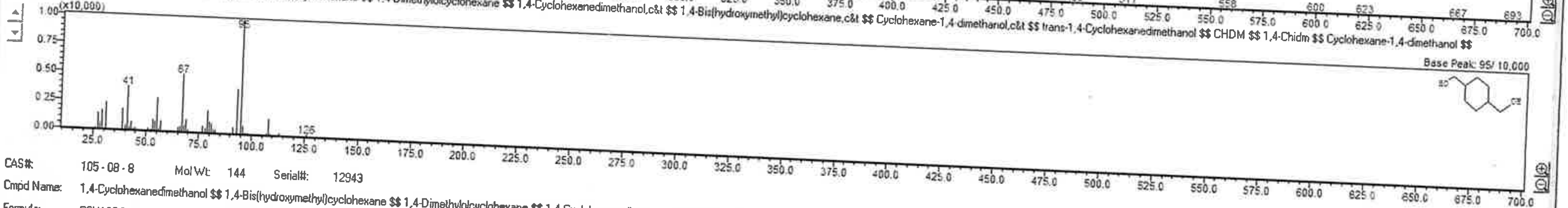

CAS#: 105 - 08 - 8 Mol Wt: 144 Serial#: 12943

Cmpd Name: 1,4-Cyclohexanedimethanol $$ 1,4-Bis(hydroxymethyl)cyclohexane $$ 1,4-Dimethylolcyclohexane $$ 1,4-Cyclohexanedimethanol,c&t $$ 1,4-Bis(hydroxymethyl)cyclohexane,c&t $$ Cyclohexane-1,4-dimethanol,c&t $$ trans-1,4-Cyclohexanedimethanol $$ CHDM $$ 1,4-Chidm $$ Cyclohexane-1,4-dimethanol $$

Formula: C8H16O2 Class Flag: No Class Flags.

Ret.Index: 1327

11:49 AM 2022/12/06
